# *PhyloPythiaS+*: a self-training method for the rapid reconstruction of low-ranking taxonomic bins from metagenomes

**DOI:** 10.7717/peerj.1603

**Published:** 2016-02-08

**Authors:** Ivan Gregor, Johannes Dröge, Melanie Schirmer, Christopher Quince, Alice C. McHardy

**Affiliations:** 1 Max-Planck Research Group for Computational Genomics and Epidemiology, Max-Planck Institute for Informatics, Saarbrücken, Germany; 2 Department of Algorithmic Bioinformatics, Heinrich-Heine-University Düsseldorf, Düsseldorf, Germany; 3 Computational Biology of Infection Research, Helmholtz Center for Infection Research, Braunschweig, Germany; 4 The Broad Institute of MIT and Harvard, Cambridge, MA, United States; 5 School of Engineering, University of Glasgow, Glasgow, United Kingdom

**Keywords:** Metagenomics, Taxonomic classification, Machine learning, Bioinformatics

## Abstract

**Background.** Metagenomics is an approach for characterizing environmental microbial communities *in situ*, it allows their functional and taxonomic characterization and to recover sequences from uncultured taxa. This is often achieved by a combination of sequence assembly and binning, where sequences are grouped into ‘bins’ representing taxa of the underlying microbial community. Assignment to low-ranking taxonomic bins is an important challenge for binning methods as is scalability to Gb-sized datasets generated with deep sequencing techniques. One of the best available methods for species bins recovery from deep-branching phyla is the expert-trained *PhyloPythiaS* package, where a human expert decides on the taxa to incorporate in the model and identifies ‘training’ sequences based on marker genes directly from the sample. Due to the manual effort involved, this approach does not scale to multiple metagenome samples and requires substantial expertise, which researchers who are new to the area do not have.

**Results.** We have developed *PhyloPythiaS+*, a successor to our *PhyloPythia(S)* software. The new (+) component performs the work previously done by the human expert. *PhyloPythiaS+* also includes a new *k*-mer counting algorithm, which accelerated the simultaneous counting of 4–6-mers used for taxonomic binning 100-fold and reduced the overall execution time of the software by a factor of three. Our software allows to analyze Gb-sized metagenomes with inexpensive hardware, and to recover species or genera-level bins with low error rates in a fully automated fashion. *PhyloPythiaS+* was compared to *MEGAN*, *taxator-tk*, *Kraken* and the generic *PhyloPythiaS* model. The results showed that *PhyloPythiaS+* performs especially well for samples originating from novel environments in comparison to the other methods.

**Availability.** *PhyloPythiaS+* in a virtual machine is available for installation under Windows, Unix systems or OS X on: https://github.com/algbioi/ppsp/wiki.

## Introduction

Metagenomics is the functional or sequence-based analysis of microbial DNA isolated directly from a microbial community of interest (Riesenfeld, Schloss & Handelsman, 2004; Kunin et al., 2008). As the cultivation conditions for most microorganisms are unknown or too complex to reproduce in the laboratory (Hugenholtz, 2002), random shotgun and amplicon-sequencing based metagenome studies have led to substantial advances in our understanding of the structure and functions of microbial communities within the last decade (Kalyuzhnaya et al., 2008; Turnbaugh et al., 2010; Hess et al., 2011; Pope et al., 2011b; Zarowiecki, 2012; Schloissnig et al., 2013; Blaser et al., 2013). The taxonomic classification or ‘binning’ of metagenome samples is often performed after sequence assembly (Peng et al., 2011; Laserson, Jojic & Koller, 2011; Boisvert et al., 2012; Namiki et al., 2012; Pell et al., 2012). This is a computationally demanding task, which for metagenome samples results in a mixture of sequence fragments of varying lengths, originating from the different microbial community members. A taxonomic binning defines ‘bins’ of sequence fragments that were assigned the same taxonomic identifier, representing draft genomes or pan-genomes of the different microbial community members. Taxonomic binning methods use sequence homology, sequence composition and similarities of contigs in read coverage or gene counts, see Dröge & McHardy (2012) for a recent review. The subsequent analysis of these bins allows characterizing the functional and metabolic potential for individual taxa. For instance, in a collaboration with Mark Morrison’s group, a functional and metabolic analysis of a draft genome recovered by taxonomic binning from the gut of the Australian Tammar Wallaby metagenome led to the isolation and subsequent characterization of a new and previously uncultivated bacterium (Pope et al., 2011b). Different from binning methods, taxonomic profiling tools (Wu & Eisen, 2008; Stark et al., 2009; Liu et al., 2011; Meinicke, Asshauer & Lingner, 2011; Wu & Scott, 2012; Segata et al., 2012; Sunagawa et al., 2013; Silva et al., 2013) return a taxonomic profile for a metagenome sample to represent the taxonomic composition of the underlying sampled community.

Composition-based binning methods assign metagenome sequences based on their *k*-mer signature, which is derived from the counts of short oligomers (*k*-mers) for a sequence (Karlin & Burge, 1995; Deschavanne et al., 1999). Our previously developed *PhyloPythia(S)* (*PPS*) (McHardy et al., 2007; Patil, Roune & McHardy, 2011) software uses this information in combination with a structured output support vector machine framework for taxonomic classification. Composition-based signatures are global genomic properties, which can be estimated from any sufficiently sized sequence sample for a taxon; e.g., for *PP(S)*, 100 kb of reference sequences for a taxon are sufficient for accurate assignment, also for low ranking taxa. Thus, no complete genome sequences of related organisms are required for assignment, which is often a limiting factor for the homology-based methods. Composition-based methods are very fast, with classification runtimes increasing linearly with the size of the sequence sample, whereas the runtime of alignment-based methods is proportional to the product of the reference collection size and the sequence sample size. As the current sequencing technologies produce Gb-sized metagenome samples (Metzker, 2010; Loman et al., 2012), scalability and computational efficiency are becoming increasingly important for computational metagenomic methods. Therefore, we have developed a fully automated taxonomic binning software, that can rapidly process large metagenome samples. *PhyloPythiaS*+ (*PPS*+) is the successor to our previously described *PPS* software and improves on it in several important ways. We provide an automated marker-gene based framework for design and creation of sample-derived structured output support vector machine models, which allows the generation of accurate sample-derived models without user intervention or expert knowledge. *PPS*+ is the first tool that combines taxonomic profiling and subsequent taxonomic composition based binning of the whole metagenome sample, which is particularly valuable for the draft genome reconstruction of taxa from deep-branching phyla. By implementation of a faster *k*-mer counting algorithm, we substantially increased its throughput to 0.5 Gb/h. *PPS*+ is distributed in a virtual machine which facilitates installation under all common operating systems and runs on inexpensive hardware available to most users.

**Figure 1:**
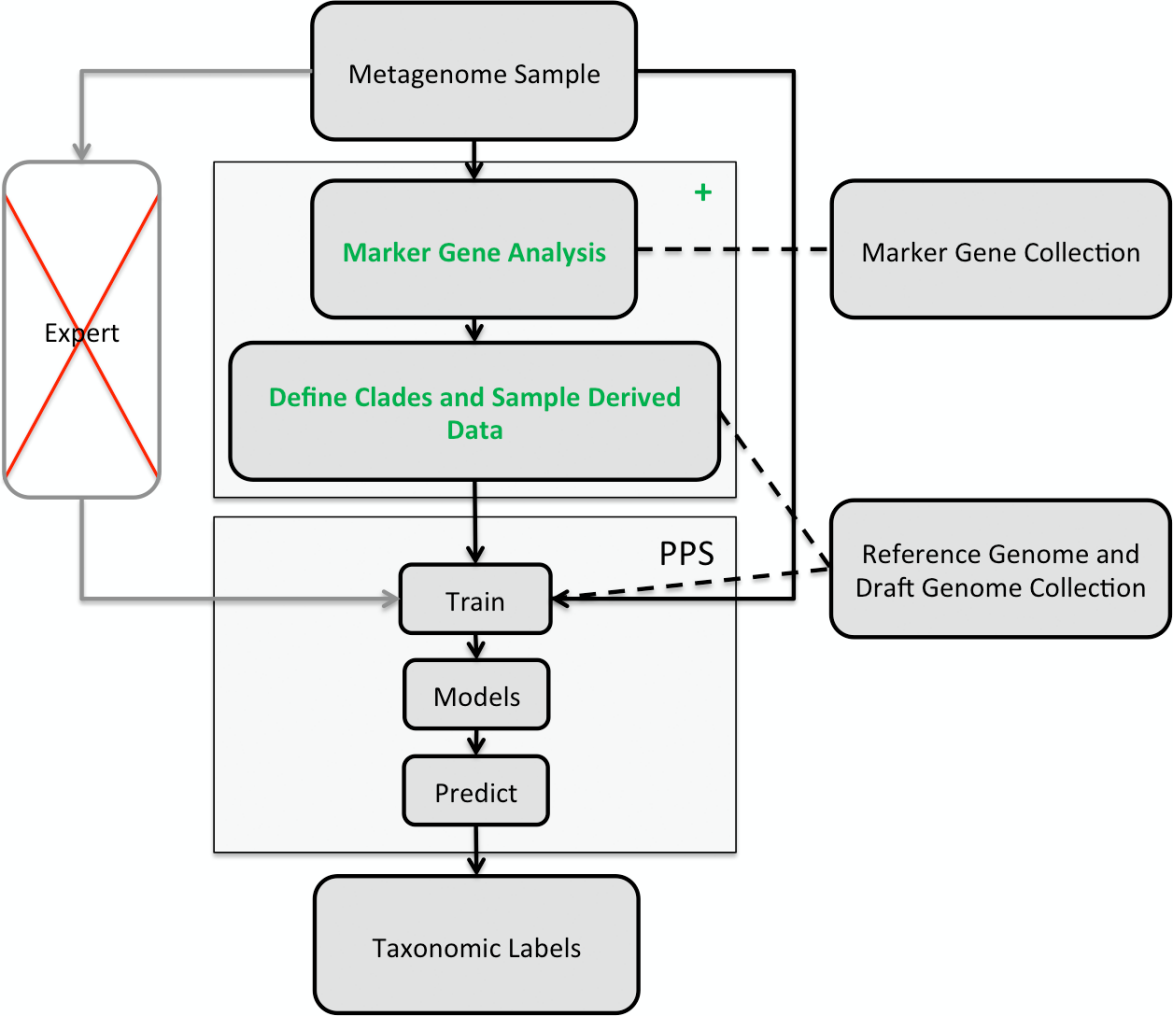
Illustration of the *PhyloPythiaS*+ workflow. The recommended use of *PPS* is that a human expert specifies the taxa to incorporate in a composition-based taxonomic metagenome classifier and identifies the relevant ‘training’ sequences based on marker genes directly from the sample. The inclusion of contigs originating directly from members of the microbial community, as ‘training’ sequences, is very important for achieving good classification accuracy, as many members of microbial communities are underrepresented in public sequence collections. In *PPS*+, the step of deciding which taxa to include in the model and defining suitable ‘training’ sequences was automated in the + component, based on marker genes, genome and draft genome sequence collections. The data generated by the + component are then used to build the *PPS* models, that are subsequently used to generate the taxonomic binning of the entire metagenome sequence sample.

## Methods

The classification of a shotgun metagenome sequence sample with *PPS*+ proceeds in two phases (Fig. 1): In the first phase, the newly developed (+) component identifies sample-derived training sequences and the taxa to be modeled by searching for copies of 34 ubiquitous taxonomic marker genes in the metagenome sample. The marker gene analysis results in taxonomic assignments for a small fraction of the sample. Based on the taxa abundance profile derived from these assignments and the sequences available in the reference sequence collections, our method determines which taxa will be modeled and which are the sample-derived data that will be used for training *PPS*.

The second phase is the composition-based taxonomic assignment of the entire metagenome sample using *PPS* models trained using the data generated in the first phase. *PPS* models can be reused to classify further metagenome samples, e.g., additional samples from the same community.

### PhyloPythiaS

Assignment with *PPS* proceeds in two steps: In the training step, an ensemble of structured output Support Vector Machines (SVMs) (Joachims, Finley & Yu, 2009) for the specified part of the NCBI taxonomy, defined by the taxa being modeled, are trained using the sample-derived training sequences and additional data for these taxa from a customized reference collection of sequenced genomes and draft genomes (Suplemental Information 1, Section 3.3). The list of modeled taxa and sample-derived data are generated with the + component of *PPS*+. The list of taxa restricts the taxonomic output space that is modeled, i.e., a sequence from a metagenome sample will be assigned to a leaf node taxon or a corresponding higher-ranking taxon of the learned taxonomy.

In the prediction step, the *PPS* model ensemble identifies the taxon which best matches a query sequence in terms of its *k*-mer profile and assigns to it the respective taxonomic identifier. By default, sequences of 1 kb or more are classified (*PPS*+ configuration parameter: *minSeqLen*).

### The + component of *PhyloPythiaS+*

The input for the + component of *PhyloPythiaS*+ is the metagenome sample. This step returns a list of clades and sample-derived data for the subsequent *PPS* training. The + component performs the following steps:

(1) *Marker gene identification*: DNA sequences from the sample are translated in all six reading frames (i.e., also considering reverse complement sequences) to protein sequences. In both the translated and untranslated sequences, regions with similarity to the DNA or protein Hidden Markov model (HMM) profiles of 34 taxonomically informative marker genes (Wu & Eisen, 2008; Stark et al., 2009; Liu et al., 2011; Wu & Scott, 2012; Segata et al., 2012; Sunagawa et al., 2013) are identified (Supplemental Information 1, Section 3.3 and 6.1). The corresponding DNA marker gene sequences from these regions are used for further analysis.
(2) *Taxonomic marker gene assignment*: The marker gene sequences are assigned a taxonomic identifier using the composition-based Naïve Bayes classifier (Schloss et al., 2009) (Supplemental Information 1, Section 6.2).
(3) *Taxonomic sequence assignment*: If a sequence contains multiple marker genes, multiple taxonomic identifiers are identified in Step 2. Then the highest bootstrap confidence score (*hcs*) returned by the Naïve Bayes classifier (NBC) for one of the markers on the fragment is identified. We use all marker gene assignments with confidence scores larger than *hcs ^∗^ (1 − candidatePlTopPercentThreshold)*. The default setting for the configuration parameter *candidatePlTopPercentThreshold* is 0.1. From the set of taxonomic identifiers, the lowest taxon *t* is identified for which all other assignments are either to the same taxon *t* or defined at higher-ranking parental taxa of *t*. Taxon *t* is consequently used for the overall fragment assignment. The confidence score for the fragment is set to the smallest confidence score for the set of retained marker gene assignments.
(4) *(Optional: Taxonomic scaffold assignment):* Scaffolding information (i.e., the mapping of contigs to scaffolds) can be used to obtain more training data for the relevant taxa. Assembled contigs can be grouped into scaffolds based on the paired-end information after the assembly. As all contigs of a particular scaffold originate from the same strain, all contigs of the respective scaffold should have the same taxonomic label. Here, we make use of this scaffolding information, such that unassigned contigs of a particular scaffold can be assigned based on the assigned contigs of the respective scaffold. In the first step, the taxonomic identifiers of all assigned contigs for a scaffold are corrected as follows: Let us consider that *n* taxonomically assigned contigs of a scaffold are placed along a common path from the root *r* down to a low-ranking clade *lc* in the reference taxonomy. The unassigned contigs of a scaffold are not among these *n* contigs. To obtain a consistent assignment for all the contigs of a scaffold and to correct for ‘outlier’ contig assignments to low ranking taxa, contigs are reassigned according to the following: All *n* assigned contigs of the respective scaffold are reassigned to the lowest taxon *c*, which lies on the path from *r* to *lc*, where *c* is chosen such that at least (*agThreshold ^∗^ n*) of the contigs are assigned on the path from *c* to *lc*. In the second step, unassigned contigs are assigned to the same taxon *c*, if a sufficient number of contigs have already been assigned. Let us denote the sum of all contig lengths for a scaffold as *l* and the sum of all assigned contig lengths of the respective scaffold as *al*. If *al/l* ≥ *assignedPartThreshold,* then the unassigned contigs of a scaffold are also assigned to clade *c* (see the configuration parameters: *placeContigsFromTheSameScaffold* = *True*, *agThreshold* = *0.3*, *assignedPartThreshold* = *0.5*).
(5) *Assignment path truncation*: Contigs assigned to a lower-ranking taxon than the specified lowest rank are reassigned to the parental taxon of this lowest rank (configuration parameter: *rankIdCut*).
(6) *Taxa selection for model specification*: Any taxon for which at least 100 kb of sample-derived data have been identified can be modeled. Furthermore, species can be modeled if at least 300 kb of reference sequences are available in the reference sequence database, and higher-ranking taxa can be modeled if data for at least three distinct species with this requirement (>300 kb per species) are available. Contigs assigned to taxa for which there are fewer data are subsequently assigned to higher taxonomic ranks for which sufficient data are available to allow their use as sample-derived training data (configuration parameters: *minGenomesWgs* = *3 or 1*, *minBpPerSpecies* = *300,000*, *minBpToModel* = *100,000*).
(7) *Abundant taxa selection*: To reduce the number of taxa to the most relevant ones, the least abundant taxon is removed iteratively. This is defined as the taxon to which the minimum number of bp is assigned. Sequences assigned to this taxon are reassigned to the closest defined taxon at a parental rank. The algorithm ends when the number of leaf taxa is less than or equal to the maximum number of taxa to be modeled (configuration parameter: *maxLeafClades* = *50*; this can be set realistically up to 800).

*Balancing training data*: The part of the taxonomy that will be modeled with *PPS* is defined by the taxa identified in the previous step. It has leaf nodes at different ranks above the specified rank cut-off, and internal nodes. Only leaf node taxa and sample-derived training data assigned to leaf node taxa in the preceding steps are specified as input for *PPS* training. To balance the training data across clades, a maximum of 400 kb of sample-derived training data are selected for each leaf node taxon (configuration parameter: *maxSSDfileSize*). For this selection, contigs are used in order of decreasing confidence values and then in order of decreasing length. The balancing of training data can be switched off by setting the configuration parameter (*maxSSDfileSize*) to a large number.

### Simultaneous counting of multiple short *k*-mers

We provide *PPS*+ with a new custom *k*-mer counting algorithm that is based on the Rabin Karp string matching algorithm (Karp & Rabin, 1987). The algorithm is highly optimized to count occurrences of short DNA sequences. It is very fast, as it is memory efficient, because it does not need any large helper data structure similar to suffix trees. It explores the locality of reference, uses very fast bit shift operations and is efficiently implemented in C. Its complexity is *O*(*n*), where *n* is the length of the DNA sequence that is being considered. It enumerates *k*-mers up to hundred times faster than when using suffix trees that were employed in *PPS*. This made *PPS*+ overall up to 3x faster than *PPS*. Because the algorithm allows to simultaneously enumerate *k*-mers of consecutive lengths in one run, it is at least 2–7x faster than the state-of-the-art software *Jellyfish* (Marcais & Kingsford, 2011) and 11x faster than *KAnalyze* (Audano & Vannberg, 2014) in the scenario used in *PPS*+, i.e., when calculating *k*-mers of length 4, 5, and 6 for every sequence (Table S1, Supplemental Information 1, Section 2). We also found that the state-of-the-art *k*-mer counting methods *KMC 2* (Deorowicz et al., 2015) and *Turtle* (Roy, Bhattacharya & Schliep, 2014) are not applicable to our problem setting, as *KMC 2* can count only *k*-mers ≥ 10 and *Turtle* is prohibitively slow for sequences ≥ 16 kb.

#### Algorithm description

Let us assume that we are given an array *a*, which represents a DNA sequence of length *n* where all letters are encoded as numbers *0, 1, 2, 3* (where *A* ∼*0, T* ∼*1, G* ∼*2, C*∼*3*) and let *a*_0_, …, *a*_*n*−1_ denote the respective entries. We would like to count the occurrences of all *k*-mers of length *k* and store the counts in an array *c* of length 4^*k*^, which is initialized by zeros. Each *k*-mer maps to a unique index in the array *c*. The index of the first *k*-mer in our sequence is calculated according to:

\begin{eqnarray*} {\mathrm{index}}_{0}={\mathrm{a}}_{0}\ast {4}^{\mathrm{k}-1}+{\mathrm{a}}_{1}\ast {4}^{\mathrm{k}-2}+\cdots +{\mathrm{a}}_{\mathrm{ k}-2}\ast {4}^{1}+{\mathrm{a}}_{\mathrm{ k}-1}\ast {4}^{0}. \end{eqnarray*}

The index of the (*i* + 1)th *k*-mer of the sequence is computed from the (*i*)th index as:

\begin{eqnarray*} {\mathrm{index}}_{i+1}=({\mathrm{index}}_{i}-{\mathrm{a}}_{i}\ast {4}^{\mathrm{k}-1})\ast {\mathrm{a}}_{i+\mathrm{k}}\ast {4}^{0}. \end{eqnarray*}

When an index is identified, the corresponding *k*-mer count at this index position in array *c* is incremented by one. For instance, the DNA sequence *ATGCATG* is encoded in array *a* as [0, 1, 2, 3, 0, 1, 2]. For *k* = 2, we would add two counts for the *k*-mer *AT* in array *c* at the index position 0∗4 + 1 = 1, two counts for *TG* at the index position 1∗4 + 2 = 6, one count for *GC* at the index position 2∗4 + 3 = 11 and one count for *CA* at index position 3∗4 + 0 = 12. The multiplication operation X∗4^m^ can be computed using the bit shift operation *X* ≪ 2∗*m*, which is usually much faster than multiplication.

#### Counting k-mers of different lengths at once

If *index_i_* is the index of the *i*th *k*-mer of length *k*, the index of the *i*th (*k* − *j*)-mer (of length *k* − *j*) can be simultaneously computed using the bit shift operation as *index_i_* ≫ (2∗*j*) (for *j* ∈ [1..*k* − 1]) and the corresponding counter at the computed index of a respective counter array of length 4^(*k*−*j*)^ is incremented. The end of a DNA sequence can be handled by adding several non-DNA characters to its end.

## Results

We evaluated *PPS*+ by comparing it to homology-based methods (*MEGAN4*, *taxator-tk)* (Huson et al., 2011; Dröge, Gregor & McHardy, 2014), the fast taxonomic binning program *Kraken* (Wood & Salzberg, 2014), the composition-based method *PhyloPythia* trained under expert guidance (a recommended but time-consuming procedure) and to a generic *PPS* model using default settings (Supplemental Information 1, Section 3.5–3.8). For a performance comparison of *PPS* to methods with prohibitive runtimes for large datasets, such as *PhymmBL* (Brady & Salzberg, 2011) and *CARMA3* (Gerlach & Stoye, 2011), and the web-based tool *NBC* (Rosen, Reichenberger & Rosenfeld, 2011) see Patil et al. (2011); Patil, Roune & McHardy (2011); Dröge, Gregor & McHardy (2014), as *PPS* has already been compared to these methods with favorable outcomes. For a comparison with ‘taxonomy-free’ binning software *CLARK* (Ounit et al., 2015) see (Supplemental Information 1, Section 7). We did not compare *PPS*+ to profiling tools such as (Liu et al., 2011), as *PPS*+ is a binning method that assigns a taxonomic label to each input sequence. As benchmark datasets, we created two simulated datasets, one with a uniform (137 Mb) and one with a log-normal (66 Mb) distribution of 47 community members (Supplemental Information 1, Section 3.1, Datasets S1 and S2). We also used two real datasets, a metagenome sample from the guts of two obese human twins (255 Mb) (Turnbaugh et al., 2010) and a cow rumen metagenome sample (319 Mb) from Hess et al. (2011) (Supplemental Information 1, Section 3.2, Datasets S3–S6) for evaluation.

### Benchmarks with simulated datasets

We constructed the simulated datasets by assembling simulated reads with an empirical error profile. The details on how the simulated reads were generated and assembled can be found in (Supplemental Information 1, Section 3.1). For the evaluation, precision and recall were calculated (Supplemental Information 1, Section 3.9). Furthermore, these measures were also calculated with a ‘correction,’ to account for the case where the sequences of one taxon were consistently assigned to a different taxon, as for draft genome reconstruction, it is more important that the sequences are assigned consistently than that the taxonomic identifier is correct. To assess the performance of the different methods in assigning the simulated sequence fragments without related reference genomes being available, ‘new strain,’ ‘new species’ and ‘new genus’ scenarios were simulated by removing all sequence data from the taxa of the simulated test dataset at each rank from the reference data. Furthermore, for *PPS*+, we distinguished whether the reference data were excluded (masked) from the reference sequence (RS) collection or also from the marker gene (*MG*) collection, since the *MG* collection included sequences for 15 times more distinct species than the RS collection. There were therefore two different situations to consider (Table 1).

**Figure 2:**
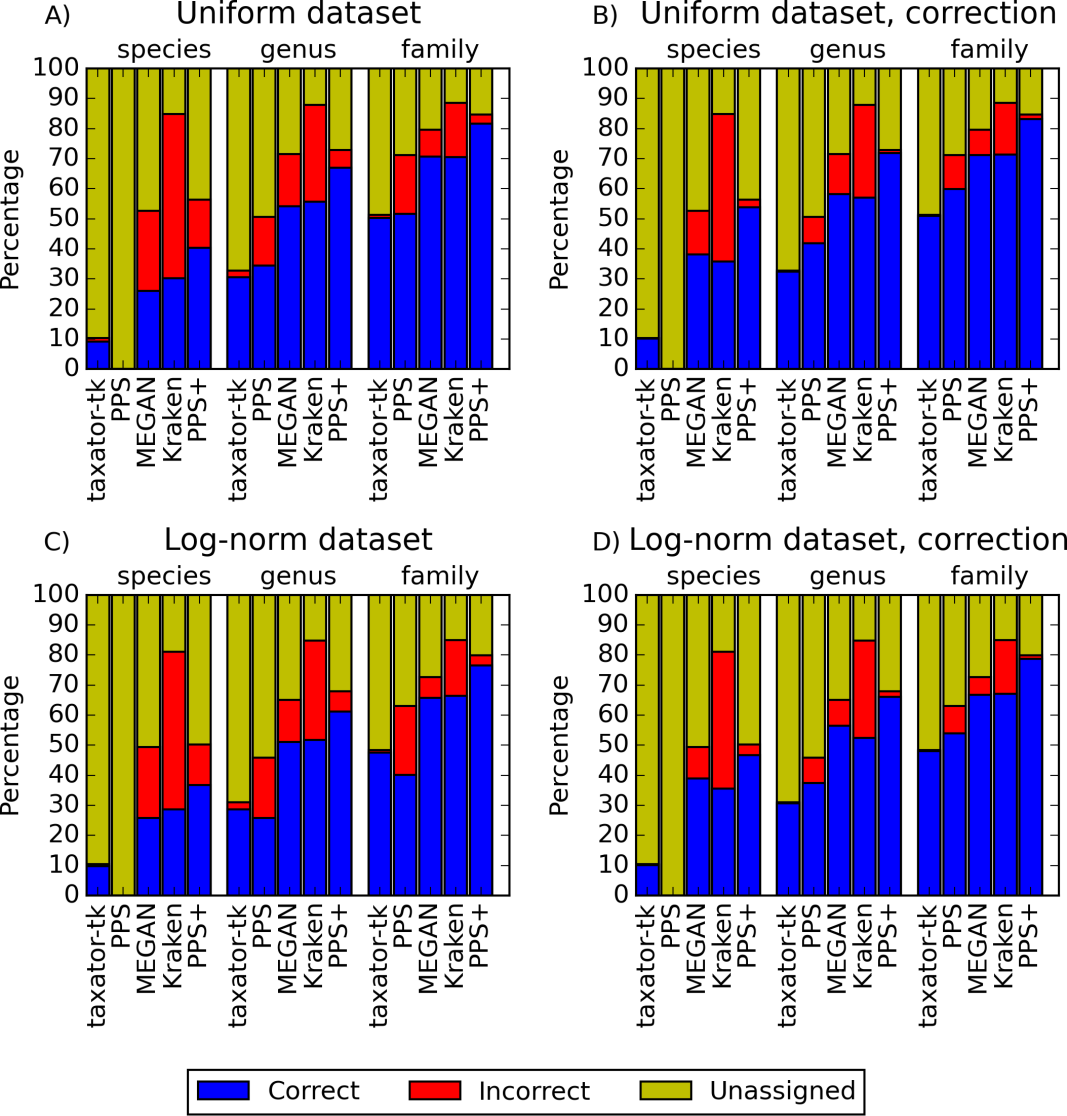
Performance comparisons with simulated datasets. (A) and (C) show the fraction of correct, incorrect and unassigned bp for simulated datasets with uniform and log-normally distributed species abundance for *PhyloPythiaS*+, the generic *PhyloPythiaS* model, *MEGAN4, Kraken* and *taxator-tk* for assignments at the species, genus and family ranks. Results were averaged over all test ‘scenarios’ (Table 1), where sequences of the same strain, species or genus from the simulated metagenomes were removed from the genome, draft genome and marker gene reference sequence collections (Figs. S1, S3, S14A and S14C). (B) and (D) show the portion of consistently (correct), inconsistently (incorrect) and unbinned (unassigned) bp without consideration of the taxonomic identifiers (Figs. S2, S4, S14B and S14D, Supplemental Information 1, Section 3.9.2). The exact values and the corresponding precision, recall and *f*_1_-score are contained in (Tables S2–S5) for (A–D), respectively.

**Table 1:** Test scenarios. Test scenarios where data was removed (masked) up to the specified rank for the corresponding taxa represented in the simulated metagenome datasets from the reference collections. RS denotes the reference collection of complete or draft genomes; *MG* indicates the reference collection of marker genes (Supplemental Information 1, Section 3.3).

Test scenario	Rank masked from RS	Rank masked from *MG*
1.	None	None
2.	Strain	None
3.	Species	None
4.	Genus	None
5.	Strain	Strain
6.	Species	Strain
7.	Genus	Strain
8.	Species	Species
9.	Genus	Genus

*PPS*+ showed a substantially improved precision and recall over the *PPS* generic model, which demonstrated the impact of the improved selection of training data and modeled taxa (Figs. 2A and 2C, S1A–S1D and S3A–S3D). *PPS*+ almost always had higher precision and recall than *MEGAN4* and *Kraken,* except when almost all test data were included in the reference sequences (Figs. 2A and 2C, S1A–S1C, S1E, S3A–S3C, S3E, S14A). This was even more pronounced when comparing bin quality using the corrected measures (Figs. 2B and 2D, S2A–S2C, S2E, S4A–S4C, S4E, S14A and S14D). When comparing *PPS*+ to *taxator-tk*, *PPS*+ had substantially improved recall, particularly for lower ranks (Figs. 2A and 2C, S1A–S1C, S1F, S3A–S3C, S3F); while *taxator-tk* outperformed all other methods in terms of precision (Figs. 2A and 2C, S1A–S1F and S3A–S3F). Both methods were similarly precise when analyzing bin recovery, independent of assigning the taxonomic identifiers to the corrected measures (Figs. 2B and 2D, S2A–S2C, S2F, S4A–S4C and S4F). As a strong point of *PPS*+ , we also observed that it more rarely predicted wrong taxa that were not a part of the sample than the other methods (Fig. S5). For example, for the genus rank in Scenarios 3 and 8, *PPS*+ assigned sequences to only 2–5 false positive taxa, while *taxator-tk* identified 20, *MEGAN4* 37 and *PPS* 59 false ones. If *PPS*+ identified wrong taxa, these were usually very closely related to the true taxa.

### Benchmarks with real datasets

#### Comparison of scaffold and contig assignments

For each taxonomic rank, the percentage and the total number of kb (% agreement and kb agreement) that were assigned the same taxonomic identifier were calculated for the real datasets, based on the assignments of scaffold and contig sequences (Supplemental Information 1, Section 3.10.1). For the chunked cow rumen dataset (Supplemental Information 1, Section 3.2.2), *taxator-tk* had the highest assignment consistency (Table 2); however, it assigned much fewer data than the other methods at lower taxonomic ranks. A detailed comparison is given in heat maps (Figs. S6–S13). *PPS*+ performed substantially better by both measures than the generic *PPS* model in almost all cases. *PPS*+ was also more consistent than *MEGAN4* for all lower ranks and assigned many more sequences than *MEGAN4* overall. For instance, at the genus rank, the scores were 84.3 and 56 ‘% agreement’, as well as 33,724 and 13,726 ‘kb agreement’ for *PPS*+ and *MEGAN4*, respectively. The overall low numbers for *Kraken* suggests that it is rather not applicable to samples containing novel taxa. Also, the low number of consistently assigned bp by *MEGAN4* and *taxator-tk* to lower taxonomic ranks reflects the availability of few related reference genome sequences for the cow rumen metagenome sample, which is not an issue for a composition-based method *PPS*+.

**Table 2:** Comparison of contig and scaffold assignments of the chunked cow rumen dataset. Contigs of the cow rumen dataset of at least 10 kb were divided into chunks of 2 kb for evaluation of assignment consistency (Supplemental Information 1, Section 3.2.2). The contigs and scaffolds of the chunked cow rumen dataset were assigned using *PPS*+ , the generic *PPS* model, *MEGAN4*, *taxator-tk* and *Kraken*. For each method, up to two taxonomic identifiers were assigned to each contig at each rank, i.e., one identifier came from the contig assignment and the second identifier came from the corresponding scaffold assignment. Contigs with less than two taxonomic assignments at each rank were not considered in this comparison. The measure ‘% agreement’ was the percentage of contigs with the same two taxonomic identifiers at a particular rank, whereas ‘kb agreement’ was the total number of kb of contigs with the same taxonomic identifiers (Supplemental Information 1, Section 3.10.1). Bold numbers correspond to the best values, whereas italic numbers indicate the worst values.

Method	Rank	% agreement	kb agreement
*PPS*+	Phylum	73.9	**153,774**
*PPS*	Phylum	67.8	75,538
*MEGAN4*	Phylum	74.2	43,380
*taxator-tk*	Phylum	**98.2**	59,702
*Kraken*	Phylum	*67.0*	*33,558*
*PPS*+	Class	86.0	**99,596**
*PPS*	Class	*58.5*	43,931
*MEGAN4*	Class	68.5	33,780
*taxator-tk*	Class	**97.7**	*23,190*
*Kraken*	Class	*58.5*	27,536
*PPS*+	Order	88.4	**98,616**
*PPS*	Order	63.8	41,349
*MEGAN4*	Order	68.9	32,650
*taxator-tk*	Order	**98.0**	*22,368*
*Kraken*	Order	*57.0*	26,410
*PPS*+	Family	80.0	**46,343**
*PPS*	Family	55.8	19,158
*MEGAN4*	Family	55.0	15,790
*taxator-tk*	Family	**98.9**	*7,276*
*Kraken*	Family	*45.2*	18,370
*PPS*+	Genus	84.3	**33,724**
*PPS*	Genus	63.2	12,938
*MEGAN4*	Genus	56.0	13,726
*taxator-tk*	Genus	**99.1**	*6,042*
*Kraken*	Genus	*43.7*	16,912
*PPS*+	Species	91.6	9,821
*PPS*	Species	N/A	N/A
*MEGAN4*	Species	54.6	8,502
*taxator-tk*	Species	**100.0**	*292*
*Kraken*	Species	*38.1*	**14,186**

For the human gut microbiome, extensive sequencing of isolate cultures has resulted in a large collection of several hundred reference genome sequences. Accordingly, for the human gut dataset, *taxator-tk*, *MEGAN4 and Kraken* assigned many more sequences than they did for the cow rumen dataset (Tables 2 and 3). For *Kraken* and *MEGAN4*, this was most pronounced for the genus and species ranks, even though this was also caused by counting scaffolds containing only one contig being consistent to itself. The most consistent method was again *taxator-tk*, but it also assigned fewer sequences than the other methods. *PPS*+ performed better than the generic *PPS* model in all cases in terms of both measures (Table 3). *PPS*+ and *MEGAN4* showed comparable consistency, with *PPS*+ being more consistent for the class, order and species ranks, and *MEGAN4* being more consistent for the superkingdom, family and genus ranks. However, *PPS*+ consistently assigned (kb agreement) more sequences than *MEGAN4*, except for the genus and species ranks. Thus, in the case of larger collections of related isolate genome sequences being available, composition- and homology-based methods perform similarly well.

**Table 3:** Comparison of contig and scaffold assignments of the human gut metagenome dataset. Contig and scaffold sequences of the human gut metagenome dataset were assigned using *PPS*+, the generic *PPS* model, *MEGAN4*, *taxator-tk* and *Kraken*. The measures ‘% agreement’ and ‘kb agreement’ were used to compare individual methods (Supplemental Information 1, Section 3.10.1). Bold numbers correspond to the best values, whereas italic numbers indicate the worst values.

Method	Rank	% agreement	kb agreement
*PPS*+	Phylum	99.0	**140,283**
*PPS*	Phylum	*97.0*	124,884
*MEGAN4*	Phylum	99.0	127,658
*taxator-tk*	Phylum	**100.0**	*104,475*
*Kraken*	Phylum	97.6	123,428
*PPS*+	Class	99.5	**134,707**
*PPS*	Class	96.9	118,068
*MEGAN4*	Class	98.5	122,131
*taxator-tk*	Class	**100.0**	*84,228*
*Kraken*	Class	*96.3*	121,071
*PPS*+	Order	99.5	**134,127**
*PPS*	Order	97.3	117,185
*MEGAN4*	Order	98.6	121,811
*taxator-tk*	Order	**100.0**	*83,337*
*Kraken*	Order	*96.3*	121,003
*PPS*+	Family	94.0	**110,664**
*PPS*	Family	92.6	97,066
*MEGAN4*	Family	96.2	98,582
*taxator-tk*	Family	**99.8**	*43,751*
*Kraken*	Family	*89.4*	109,151
*PPS*+	Genus	95.3	82,992
*PPS*	Genus	91.9	58,883
*MEGAN4*	Genus	96.1	86,495
*taxator-tk*	Genus	**99.9**	*34,667*
*Kraken*	Genus	*88.3*	**97,097**
*PPS*+	Species	94.7	43,329
*PPS*	Species	N/A	N/A
*MEGAN4*	Species	93.5	64,554
*taxator-tk*	Species	**99.7**	*10,314*
*Kraken*	Species	*81.3*	**94,591**

The taxonomic scaffold-contig consistency of the assignments was additionally evaluated (Table S6 and Table S7) using a set of measures (Supplemental Information 1, Section 3.10.2) that provide more detailed insights into assignment consistency (Supplemental Information 1, Section 5.1) and support the conclusions in this section.

#### Comparison to an expert binning based on marker genes

**Figure 3:**
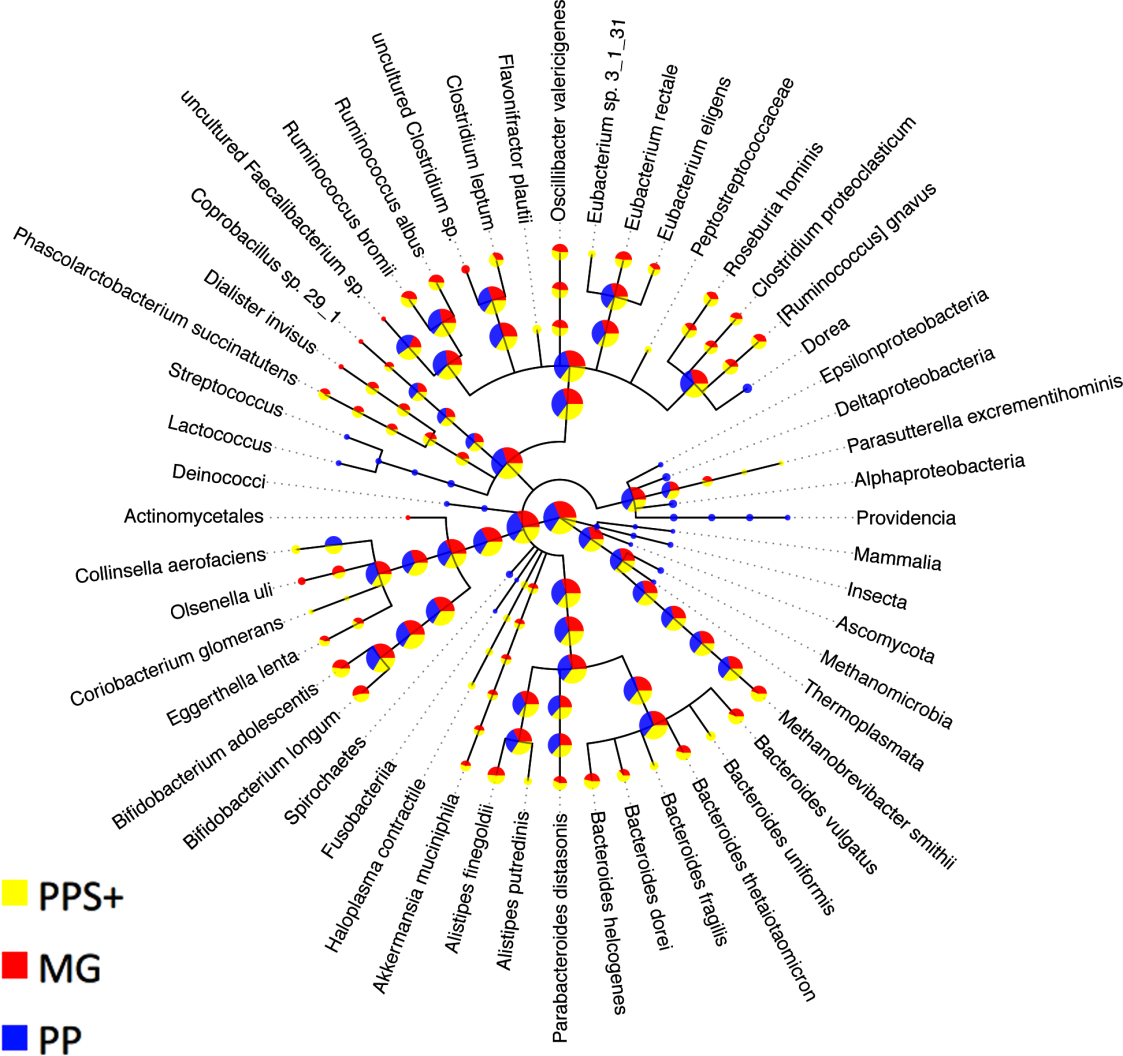
Comparison to expert binning based on marker genes. The amount of assigned bp by *PhyloPythia* (*PP*), *PhyloPythiaS*+ (*PPS*+) and taxonomically informative marker genes directly (*MG*) to each taxon are indicated by the pie chart sizes on a log-scale for the human gut metagenome sample (Turnbaugh et al., 2010; Patil, Roune & McHardy, 2011). *PhyloPythiaS*+ automatically determined the taxa to model from the sample. For the expert-trained *PhyloPythia*, the taxa to model were specified by an expert, and were included in the model if they were covered by sufficient reference sequence data retrieved separately from the sample and from sequenced human gut isolates. *PhyloPythiaS*+ assigned sequences to low-ranking taxa down to the species level, in agreement with the marker gene assignments, while *PhyloPythia* often assigned these sequences to the parental taxa. For the *MG* assignments, a negligible amount—only two contigs (3.6 kb) of two scaffolds (231 kb)—were used as sample-derived training data for *PPS*+; as mainly sample contigs (2.5 Mb) that were not part of scaffolds were used as sample-derived data to train *PPS*.

A taxonomic binning generated by *PhyloPythia* (*PP*) with expert guidance for sample-derived model construction (Turnbaugh et al., 2010) was compared to the *PPS*+ assignments. Scaffolds that were unassigned by either method were not considered. The *PP* expert binning and the *PPS*+ binning agreed well, down to the order rank (Table 4). For the family and genus ranks, the overlap of both methods dropped to 69.5–74.1%, which may partly be due to changes in the NCBI taxonomy since the generation of the expert binning in 2009. Both *PPS*+ and *PP* assignments were highly consistent with the *MG* assignments made by the + component of *PPS*+ alone, though only a small number of scaffolds with marker genes could be compared (7–23% for different ranks). While *PPS*+ had a larger overlap (‘% agreement’) with the *MG* assignments at the genus rank, *PP* had a larger overlap (‘% agreement’) with the *MG* assignments at the family rank. Moreover, we compared the number of taxonomic assignments for individual methods (Fig. 3): *PPS*+ assigned sequences to low-ranking taxa down to the species level, in agreement with the *MG* assignments, while *PP* often assigned the respective sequences only to the parental taxa. This demonstrates that *PPS*+ can generate a high quality taxonomic binning in a fully automated manner.

**Table 4:** Comparison to an expert binning based on marker genes. Comparison of the taxonomic assignments of *PPS*+ versus *PhyloPythia* (*PP*), with expert guidance for sample-derived model construction (Turnbaugh et al., 2010) for the human gut scaffolds (161, 343 kb) based on marker genes (*MG*), using the + component of *PPS*+. The measure ‘% agreement’ represents the percentage of bp assigned by both methods to the same taxonomic identifiers at a given rank, whereas ‘kb agreement’ is the corresponding number of kb assigned by both methods to the same taxonomic identifier. Scaffolds assigned by only one method are not considered in this comparison. Bold numbers correspond to the best values, whereas italic numbers indicate the worst values.

Comparison	Rank	% agreement	kb agreement
*PP vs PPS*+	Superkingdom	99.6	**160,617**
*MG vs PP*	Superkingdom	**99.7**	38,314
*MG vs PPS*+	Superkingdom	*99.5*	*38,220*
*PP vs PPS*+	Phylum	*95.4*	**149,213**
*MG vs PP*	Phylum	96.9	*17,771*
*MG vs PPS*+	Phylum	**98.7**	18,065
*PP vs PPS*+	Class	*97.0*	**145,887**
*MG vs PP*	Class	98.1	*17,599*
*MG vs PPS*+	Class	**100.0**	17,869
*PP vs PPS*+	Order	*98.0*	**145,373**
*MG vs PP*	Order	98.3	*17,494*
*MG vs PPS*+	Order	**100.0**	17,764
*PP vs PPS*+	Family	*69.5*	**95,779**
*MG vs PP*	Family	**90.7**	13,047
*MG vs PPS*+	Family	83.7	*12,013*
*PP vs PPS*+	Genus	*74.1*	**78,686**
*MG vs PP*	Genus	91.6	12,235
*MG vs PPS*+	Genus	**94.9**	*11,479*

### Throughput comparison

**Figure 4:**
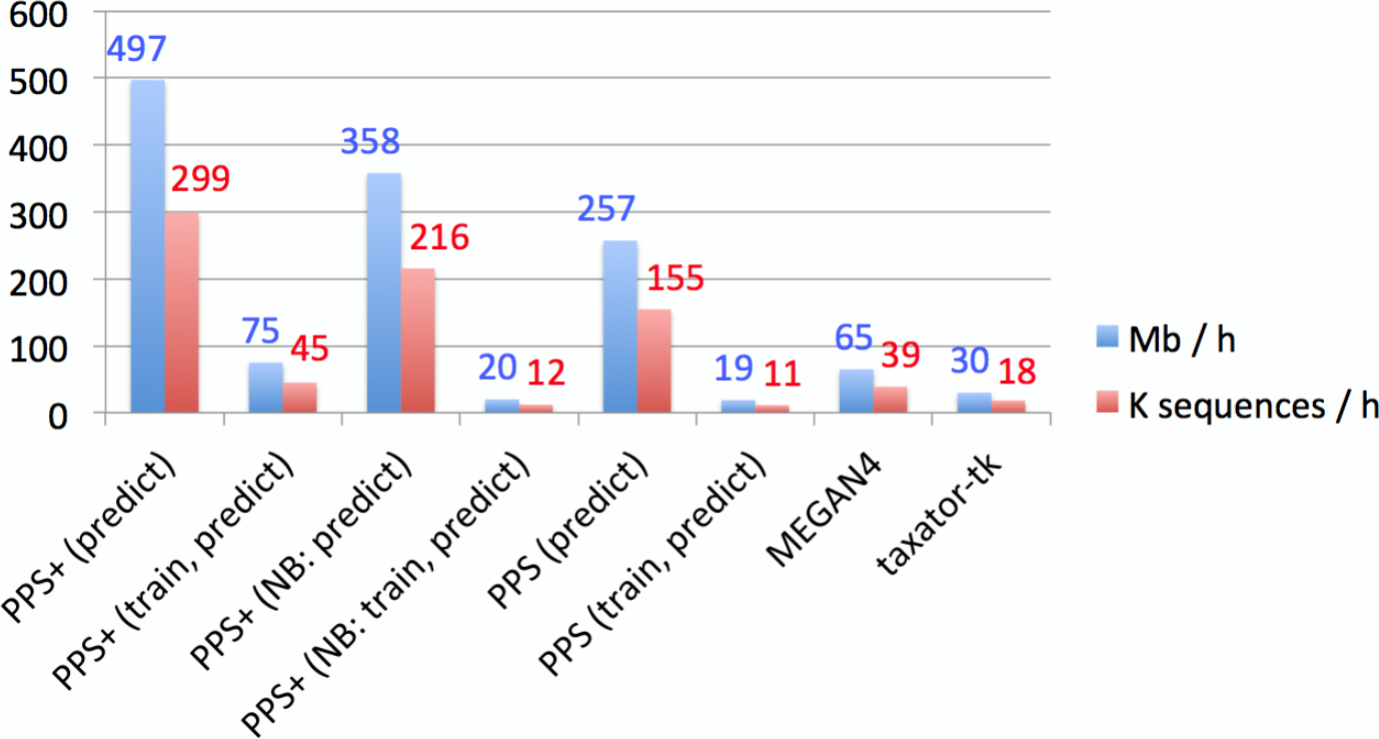
Empirical comparison of execution times. The throughput was measured in Mb and the number of sequences classified within 1 h with one execution thread, using all assembled contigs of the human gut metagenome dataset on a server computer with an AMD Opteron 6386 SE 2.8 GHz processor and 512 GB of RAM. Default settings were used for all methods (Supplemental Information 1, Section 3.5–3.7). Both *MEGAN4* and *taxator-tk* were run using *BLAST*. For *MEGAN4*, only the runtime of *BLAST* was considered, as the runtime of the subsequent algorithm was negligible. For *PhyloPythiaS* and *PhyloPythiaS*+, the throughput was calculated for the prediction step and both steps (training and prediction). The former is relevant when using previously generated models for the classification of multiple samples. The execution time shown for *PhyloPythiaS* is approximately three times better than that for the original release, as we incorporated the new *k*-mer counting algorithm. *PhyloPythiaS*+ was the only method that could also be executed on a standard laptop (NB) with an Intel i5 M520 2.4 GHz processor, 4 GB of RAM and 150 GB disk space.

The throughput of the individual methods for contig assignments of the human gut sample was calculated (Supplemental Information 1, Section 3.3, 3.4 and 5.3). The throughput of *Kraken* substantially varied between 38.4 Mb/h and 4.2 Gb/h in our experiments, depending on whether its large (∼200 GB) reference database was already loaded in the main memory or not, therefore *Kraken* is the fastest method in high performance environments. When only the prediction step of *PPS*+ was considered, *PPS*+ assigned up to 0.5 Gb/h and was more than 7 times faster than the homology-based methods (Fig. 4). This is relevant when *PPS* models are reused for the classification of another sample. Moreover, unlike the homology-based tools and *Kraken*, *PPS*+ can be run on a standard laptop, as it requires much less main memory (see Supplemental Information 1, Section 3.4 for the hardware configurations used).

## Conclusions

We describe a taxonomic assignment program that produces accurate assignments with a precision of 80% or more also for low-ranking taxa from metagenome samples. *PPS*+ is a fully automated successor of the *PhyloPythiaS* software. It automatically determines the most relevant taxa to be modeled and suitable training sequences directly from the input sample, which are then used to generate a sample-specific structured output SVM taxonomic classifier for the taxonomic binning of a sample. This enables its use for researchers without experience in the field or time to search for suitable training sequences for the manual construction of well-matching taxonomic classifier to a particular metagenome sequence sample.

*PPS*+ is best suited for the analysis of large NGS metagenome samples with assembled contigs (> 1kb) carrying marker genes or datasets including the high quality longer PacBio (Chin et al., 2013) consensus reads. Contrary to some recent methods for the taxonomic profiling or binning of multiple similar samples (Sunagawa et al., 2013), *PPS*+ can be also applied to individual samples. *PPS*+ requires only 100 kb of sample-derived data to model a bin, while homology-based methods require large related reference genome or draft genome sequence collections for substantial assignments to low-ranking taxa. Our experiments on both real and simulated metagenome samples showed that *PPS*+ automatically reconstructed many low-ranking bins from metagenome samples, such as for genera and species, representing draft genomes or pan-genomes of different community members.

The novel implementation of the *k*-mer counting algorithm accelerated *k*-mer counting 100-fold in comparison to the original *PPS* software and made *PPS*+ overall up to three times faster. The method performed favorably in comparison to all state-of-the-art *k*-mer counting software for the simultaneous enumeration of 4–6-mers, commonly used for composition-based binning.

*PPS* models can be reused when classifying multiple samples from the same or similar environments. When comparing assignment with *PPS*+ to *MEGAN4* and *taxator-tk*, *PPS*+ showed a competitive processing time, allowing to process up to 0.5 Gb of sequences per hour with a given *PPS* model on a single core with much lower main memory requirements, while *MEGAN4* processed 0.065 Gb and *taxator-tk* 0.03 Gb (Fig. 4). The fastest method in the comparison was *Kraken* with up to 4.2 Gb/h; however, we have found that *Kraken* should be used only for well-studied environments, for which many closely related (draft) genomes have been sequenced, as an alternative to alignment-based methods, as its use for samples originating from novel environments is very limited (Fig. 2).

In terms of assignment quality, we found that *PPS*+ often outperformed *MEGAN4* and *Kraken* in terms of precision, recall and consistency. *Taxator-tk* performed best in terms of precision and consistency, but assigned substantially fewer sequences to low taxonomic ranks. *PPS*+ also excelled in determining the taxa that were part of the simulated metagenome community. We found that the fully automated *PPS*+ binning can be as good as an expert-guided binning with the original *PhyloPythia* implementation. *PPS*+ also showed a substantially improved assignment performance compared to the generic *PPS* model.

To conclude, the newly introduced self-training (+) component and the faster *k*-mer counting algorithm implemented in *PPS*+ allow users to generate high quality taxonomic binnings of metagenome samples in a high-throughput fashion, without requiring expensive hardware, manual intervention and expert knowledge. It should be helpful to a wide range of users. An initial version of the software has been already employed for the taxonomic binning of a metagenome sample from reindeer guts by Pope et al. (2011a) and it is currently used in several other projects: for instance, a *PPS*+ binning of shotgun metagenome samples indicated the likely metabolite flow and participating microbial phylotypes for a biogas-producing microbial community tolerant of high ammonia levels (Supplemental Information 2).

*PPS*+ is distributed with a large reference sequence collection (containing Bacterial and Archaeal data) in a virtual machine, which makes it easy to install. This allows metagenome sample analysis on a standard laptop under Windows, Unix or OS X systems.

## Supplemental Information

10.7717/peerj.1603/supp-1 Text S1 Supplemental Text S1

10.7717/peerj.1603/supp-2 Text S2 Supplemental Text S2 Personal communication from Dr PB Pope.

10.7717/peerj.1603/supp-3 Figure S1 Benchmark results for the simulated dataset with uniform distribution Precision (P) and recall (R) (Supplemental Information 1, Section 3.9.1) at different taxonomic ranks were calculated for (A–C) *PPS*+, (D) the generic *PPS* model, (E) *MEGAN4* and (F) *taxator-tk* in all test scenarios (Table 1: Test Scenarios 1–9). In parentheses, (mg) and (rs) denote whether the sequences at a given taxonomic rank were masked from the marker gene or from the reference sequence collections, respectively (Supplemental Information 1, Sections 3.1 and 3.3). If not stated, sequences were masked from both reference collections.

10.7717/peerj.1603/supp-4 Figure S2 Benchmark results for the simulated dataset with uniform distribution using ‘correction’ Precision (P) and recall (R) were calculated with a ‘correction’ (Supplemental Information 1, Section 3.9) at different taxonomic ranks for (A–C) *PPS*+, (D) the generic *PPS* model, (E) *MEGAN4* and (F) *taxator-tk* in all test scenarios (Table 1: Test Scenarios 1–9, Supplemental Information 1, Section 3.1).

10.7717/peerj.1603/supp-5 Figure S3 Benchmark results for the simulated dataset with the log-normal distribution Precision (P) and recall (R) (Section 3.9.1) at different taxonomic ranks were calculated for (A–C) *PPS*+, (D) the generic *PPS* model, (E) *MEGAN4* and (F) *taxator-tk* in all test scenarios (Table 1: Test Scenarios 1–9, Supplemental Information 1, Section 3.1).

10.7717/peerj.1603/supp-6 Figure S4 Benchmark results for the simulated dataset with the log-normal distribution using ‘correction’ Precision (P) and recall (R) were calculated with a ‘correction’ (Supplemental Information 1, Section 3.9) at different taxonomic ranks for (A–C) *PPS*+, (D) the generic *PPS* model, (E) *MEGAN4* and (F) *taxator-tk* in all test scenarios (Table 1: Test Scenarios 1–9, Supplemental Information 1, Section 3.1).

10.7717/peerj.1603/supp-7 Figure S5 Base pairs assigned to individual taxa for a simulated metagenome of a microbial community with log-normally distributed species abundance The number of taxonomic assignments to each taxon in bp is indicated on a log-scale by the pie chart sizes for *PPS*+, the generic *PPS* model, *taxator-tk*, *MEGAN4* and the underlying standard of truth (TRUE). There were 47 strains present in the simulated metagenome sample. Assignments to taxa not shown in black in the chart are to false taxa that are not present in the simulated metagenome. (A) shows the scenario where sequences from the same species as those of the simulated dataset were excluded from the reference sequences but not the marker gene databases (Table 1: Test Scenario 3). (B) shows the scenario where sequences from the same species as those of the simulated dataset were excluded from the reference sequence and marker gene databases (Table 1: Test Scenario 8).

10.7717/peerj.1603/supp-8 Figure S6 Comparison of scaffold and contig assignments using *PPS*+ for the chunked cow rumen dataset The comparisons were performed at different taxonomic ranks using heat maps (Supplemental Information 1, Sections 3.2.2 and 3.10.1). The rows correspond to scaffolds and the columns correspond to contig assignments. (A) Phylum; (B) class; (C) order; (D) family; (E) genus; (F) species.

10.7717/peerj.1603/supp-9 Figure S7 Comparison of scaffold and contig assignments using the generic *PPS* model for the chunked cow rumen dataset The comparisons were performed at different taxonomic ranks using heat maps (Supplemental Information 1, Sections 3.2.2 and 3.10.1). The rows correspond to scaffolds and the columns correspond to contig assignments. (A) Phylum; (B) class; (C) order; (D) family; (E) genus.

10.7717/peerj.1603/supp-10 Figure S8 Comparison of scaffold and contig assignments using *MEGAN4* for the chunked cow rumen dataset The comparisons were performed at different taxonomic ranks using heat maps (Supplemental Information 1, Section 3.2.2 and 3.10.1). The rows correspond to scaffolds and the columns correspond to contig assignments. (A) Phylum; (B) class; (C) order; (D) family; (E) genus; (F) species.

10.7717/peerj.1603/supp-11 Figure S9 Comparison of scaffold and contig assignments using *taxator-tk* for the chunked cow rumen dataset The comparisons were performed at different taxonomic ranks using heat maps (Supplemental Information 1, Section 3.2.2 and 3.10.1). The rows correspond to scaffolds and the columns correspond to contig assignments. (A) Phylum; (B) class; (C) order; (D) family; (E) genus; (F) species.

10.7717/peerj.1603/supp-12 Figure S10 Comparison of scaffold and contig assignments using *PPS*+ for the human gut dataset The comparisons were performed at different taxonomic ranks using heat maps (Supplemental Information 1, Sections 3.2.1 and 3.10.1). The rows correspond to scaffolds and the columns correspond to contig assignments. (A) Phylum; (B) class; (C) order; (D) family; (E) genus; (F) species.

10.7717/peerj.1603/supp-13 Figure S11 Comparison of scaffold and contig assignments using the generic *PPS* model for the human gut dataset The comparisons were performed at different taxonomic ranks using heat maps (Supplemental Information 1, Sections 3.2.1 and 3.10.1). The rows correspond to scaffolds and the columns correspond to contig assignments. (A) Phylum; (B) class; (C) order; (D) family; (E) genus.

10.7717/peerj.1603/supp-14 Figure S12 Comparison of scaffold and contig assignments using *MEGAN4* for the human gut dataset The comparisons were performed at different taxonomic ranks using heat maps (Supplemental Information 1, Sections 3.2.1 and 3.10.1). The rows correspond to scaffolds and the columns correspond to contig assignments. (A) Phylum; (B) class; (C) order; (D) family; (E) genus; (F) species.

10.7717/peerj.1603/supp-15 Figure S13 Comparison of scaffold and contig assignments using *taxator-tk* for the human gut dataset The comparisons were performed at different taxonomic ranks using heat maps (Supplemental Information 1, Sections 3.2.1 and 3.10.1). The rows correspond to scaffolds and the columns correspond to contig assignments. (A) Phylum; (B) class; (C) order; (D) family; (E) genus; (F) species.

10.7717/peerj.1603/supp-16 Figure S14 Benchmark results for the simulated datasets with the *Kraken* software Precision (P) and recall (R) (Supplemental Information 1, Section 3.9) at different taxonomic ranks were calculated for the *Kraken* software in four test scenarios (Table 1: Test Scenarios 1, 5, 8, 9, Supplemental Information 1, Section 3.1) using the simulated datasets with the uniform (A and B) and log-norm (C and D) distribution.

10.7717/peerj.1603/supp-17 Table S1 Runtime comparison of the *k*-mer counting algorithms *Jellyfish, KAnalyze,* and the new *k*-mer counting algorithm implemented in *PPS*+ The relevant combination of *k*-mers that is typically counted for taxonomic binning is marked in bold. The benchmark was run in one thread on a server with an Intel Xeon (CPU X5660, 2.8 GHz) processor, nevertheless we observed that *Jellyfish* 1.1.1 took approximately 30% more CPU resources than specified. Parallel runs of the methods can be done by splitting the input FASTA file, running multiple instances of a tool for each file separately in parallel and merging of the result files, thus the runtimes scale approximately linearly with the number of CPUs used. As a benchmark dataset, concatenated contigs from (Turnbaugh et al., 2010) (255 Mb) were used.

10.7717/peerj.1603/supp-18 Table S2 Exact values corresponding to (Fig. 2A)

10.7717/peerj.1603/supp-19 Table S3 Exact values corresponding to (Fig. 2B)

10.7717/peerj.1603/supp-20 Table S4 Exact values corresponding to (Fig. 2C)

10.7717/peerj.1603/supp-21 Table S5 Exact values corresponding to (Fig. 2D)

10.7717/peerj.1603/supp-22 Table S6 Scaffold-contig consistency of the chunked cow rumen dataset. Contigs of the cow rumen dataset of at least 10 kb were divided into chunks of 2 kb for evaluation of assignment consistency (Supplemental Information 1, Section 3.2.2). Scaffold-contig consistency of the assignments made by *PPS*+, the generic *PPS* model, *MEGAN4*, *Kraken* and *taxator-tk* for the chunked cow rumen dataset, computed via different definitions (Supplemental Information 1, Section 3.10.2). The table also contains the number of kb of contigs assigned at low taxonomic ranks (family, genus and species) and the corresponding consistency (% agreement) (Supplemental Information 1, Section 3.10.1). Bold numbers correspond to the best values, whereas italic numbers indicate the worst values.

10.7717/peerj.1603/supp-23 Table S7 Scaffold-contig consistency of the human gut metagenome dataset Scaffold-contig consistency of the assignments made by *PPS*+, the generic *PPS* model, *MEGAN4*, *Kraken* and *taxator-tk* of the human gut dataset (Supplemental Information 1, Section 3.2.1) computed using different definitions (Supplemental Information 1, Section 3.10). Bold numbers correspond to the best values, whereas italic numbers indicate the worst values.

10.7717/peerj.1603/supp-24 Dataset S1 Simulated and real datasets used for the benchmarks

## Additional Information and Declarations

### Data Availability

The following information was supplied regarding data availability: All the external resources can be found at: https://github.com/algbioi/ppsp/wiki.
