# Supplementary material for: PhyloPythiaS+: a self-training method for the rapid reconstruction of low-ranking taxonomic bins from metagenomes
Source: PeerJ. 2016 Feb 8;4:e1603. doi: 10.7717/peerj.1603 (PMC4748697; doi:10.7717/peerj.1603)
Supplement: Text S1 [file peerj-04-1603-s001.pdf]

Supplemental Text S1

PhyloPythiaS+: A Self-Training Method for the Rapid Reconstruction of Low-Ranking Taxonomic Bins from Metagenomes

Table of Contents

1 Extended abstract 2
2 The evaluation of the *k*-mer counting algorithms 4
3 Benchmark settings 5
3.1 Simulated datasets details and generation 5
3.2 Real datasets 7
3.2.1 Human gut dataset 7
3.2.2 Cow rumen dataset 8
3.3 Reference data 8
3.4 Test environments 9
3.5 *MEGAN4* configuration 9
3.6 *Taxator-tk* configuration 10
3.7 *PPS+* and *PPS* generic model configurations 12
3.8 *Kraken* configuration 12
3.9 Assignment quality measures 13
3.9.1 Micro-averaged precision and recall 13
3.9.2 Taxonomic assignment correction for assessment of bin quality 14
3.10 Scaffold-contig consistency definitions 14
3.10.1 Comparison of scaffold and contig assignments 14
3.10.2 Taxonomic scaffold-contig assignment consistency 15
4 Detailed results for the simulated datasets 17
4.1 Uniform dataset 18
4.2 Log-normal dataset 19
4.3 Benchmarks with corrections 20
5 Detailed results for the real datasets 21
5.1 Taxonomic scaffold-contig assignment consistency 21
5.2 Evaluation summary 22
5.3 Throughput comparison 22
6 External tools 23
6.1 HMMER 3 23
6.2 MOTHUR 23
7 Evaluation of the *CLARK* software 23
8 References 25

1 Extended abstract

Metagenomics is an approach for characterizing environmental microbial communities *in situ,* it allows their functional and taxonomic characterization and to recover sequences from uncultured taxa. A major aim is to reconstruct (partial) genomes for individual community members from metagenomes. For communities of up to medium diversity (e.g. excluding environments such as soil), this is often achieved by a combination of sequence assembly and binning, where sequences are grouped into 'bins' representing taxa of the underlying microbial community from which they originate. If sequences can only be binned to higher-ranking taxa than strain or species, these bins offer less detailed insights into the underlying microbial community. Therefore, assignment to low-ranking taxonomic bins is an important challenge for binning methods as is scalability to Gb-sized datasets generated with deep sequencing techniques. Due to the importance of a match of the training data to the test dataset in machine learning for achieving high classification accuracy, one of the best available methods for the recovery of species bins from an individual metagenome sample (Patil et al., 2011; Pope et al., 2011) is the expert-trained *PhyloPythiaS* package, where a human expert identifies the 'training' sequences directly from the sample using marker genes and contig coverage information and based on data availability decides on the taxa to incorporate into the composition-based taxonomic model. The sequences of a metagenome sample are consequently assigned to these or higher ranking taxa by *PhyloPythiaS*. Because of the manual effort involved, this approach does not scale to multiple metagenome samples and requires substantial expertise, which researchers who are new to the area may not have. Other methods for draft genome reconstruction use multiple related metagenome samples as input (Albertsen et al., 2013; Imelfort et al., 2014) or are not distributed as a software package (Iverson et al., 2012).

With these challenges in mind, we have developed *PhyloPythiaS+*, a successor to our previously described method *PhyloPythia(S)* (McHardy et al., 2007; Patil et al., 2011). The newly developed + component performs the work of the human expert. It screens the metagenome sample for sequences carrying copies of one of 34 taxonomically informative marker genes (Wu & Scott, 2012) (Section 3.3). Identified marker genes are taxonomically classified using an extensive reference gene collection. The + component then decides which taxa to incorporate into the composition-based taxonomic model based on the amount of

available sequence data identified from the metagenome sample, genome and draft genome reference sequence collections (Figure 1).

We evaluated *PhyloPythiaS+* on metagenome datasets of assembled simulated reads with Illumina GAII error profiles generated from a log-normal or uniform abundance distribution over 47 strains, and two real metagenome datasets from human gut and cow rumen samples (Tables 2–3, S6–S7, Sections 3). *PhyloPythiaS+* had substantially higher overall precision and recall than the generic *PhyloPythiaS* model, because of the better match of the composition-based taxonomic model to the sequenced microbial community (Figs 2 and S1–S4, Section 3.9). It performed similarly well to an expert-trained *PhyloPythia* model without requiring manual effort (Figure 3, Table 4). Comparisons to sequence-similarity-based methods such as the popular MEtaGenome ANalyser (*MEGAN*, version 4) (Huson et al., 2011) and our own *taxator-tk* (Dröge, Gregor & McHardy, 2014) software showed a substantial increase in correct assignments to low taxonomic ranks for *PhyloPythiaS+,* while maintaining acceptably low error rates (Figs 2 and S1–S5). The largest improvement in comparison to the other methods was observed for taxa from deep-branching lineages, such as from genera or families without sequenced genomes but with marker gene data for the strain or species available (Fig. S1–S4, Table 1: Test Scenarios 2–4). This is currently the case for 39,201 species represented in our 16S reference gene collection.

PhyloPythiaS+ includes a new *k*-mer counting algorithm based on the Rabin Karp string matching algorithm. The algorithm accelerated *k*-mer counting 100-fold and reduced the overall execution time of the software by a factor of three in comparison to the original *PhyloPythiaS* release (Figure 4). We found that 500 and 360 Mb/hour could be assigned by *PhyloPythiaS+* on a single CPU core of a standard compute server and a laptop, respectively. Our software thus allows to analyze Gb-sized metagenomes with inexpensive hardware, and to recover species or genera-level bins with low error rates in a fully automated fashion. *PhyloPythiaS+* is distributed in a virtual machine and is easy to install for all common operating systems.

2 The evaluation of the *k*-mer counting algorithms

The main advantage of our method is that we do not use additional helper data structures such as suffix trees, since we work directly with arrays that represent DNA sequences. The only larger data structure that is necessary is a one-dimensional array that contains the counts of individual *k*-mers. The algorithm also processes one sequence at a time and thus there is no need to store all the sequences in the main memory, which makes the algorithm memory-efficient (e.g. less than one MB of the main memory in the scenario used in *PPS+*). To compute the next index from a previous index, we need to perform only two bit shift operations, one addition, one subtraction and one read operation (of a_{i+k}). This ensures complexity *O(n),* where *n* is the length of the DNA sequence that is being considered.

Our *k*-mer counting algorithm was compared to *Jellyfish* (version 1.1.1), *Jellyfish* (version 2.2) (Marcais & Kingsford, 2011) and *KAnalyze* (version 0.9.7) (Audano & Vannberg, 2014) (Table S1). All programs were run for *k*-mers $k ∈ [4, … , 9].

Jellyfish (version 1.1.1) was run with default parameters as:
jellyfish count -m $k -c 3 -s 10000000 -t 1 --both-strands -o OUTPUT.txt INPUT.fasta

Jellyfish (version 2.2.) was run with the following parameters, as this yielded better runtimes as the default parameters:
jellyfish count -m $k -c 16 -s 1000000 --both-strands -o OUTPUT.txt INPUT.fasta

KAnalyze (version 0.9.7) was run as:
count -k $k -d 1 -f fasta -r -o OUTPUT.txt INPUT.fasta

Our *k*-mer counting algorithm was run as:
fasta2kmers -i INPUT.fasta -f OUTPUT.txt -j $k -k $k
However, for the simultaneous counting of *k*-mers 4, 5, and 6, the program was run as:
fasta2kmers -i INPUT.fasta -f OUTPUT.txt -j 4 -k 6

3 Benchmark settings

3.1 Simulated datasets details and generation

Our simulated mock community comprised 47 strains from 45 different species (37 different genera) defined at all major taxonomic ranks, i.e. at superkingdom, phylum, class, order, family, genus and species rank. Two simulated datasets were generated with different abundance profiles, one with a uniform distribution and one with a log-normal distribution (μ=1, σ=2).

A custom read simulator was used which utilizes position- and nucleotide-specific substitution patterns derived from experimental datasets. This allowed us to generate reads with more realistic error profiles than we would with read simulators such as *pIRS* (Hu et al., 2012), *ART* (Huang et al., 2012) or *MetaSim* (Richter et al., 2007). Furthermore, we could thus specify and test different species abundance distributions for the microbial community and generate very large datasets due to the parallelization of the simulation program. We did not use the simulated datasets from Mavromatis *et al.* (Mavromatis et al., 2007), as these are substantially smaller than the current metagenome datasets.

Both simulated datasets were generated based on Illumina GAII error profiles where the standard library preparation method was used. The insert size distribution was also based on the experimental dataset. For each dataset, 15 million paired-end reads of 90 bp were generated with an average insert size of 291 bp. The first 10 bp of the 100 bp reads in the experimental dataset were trimmed because of fluctuations in the nucleotide distributions at the starting positions, which indicated partial remains of the barcode sequence. The read simulator produces output in FASTA format, which was converted into a pseudo-FASTQ format for the downstream analysis with uniformly high quality scores. The reads were then assembled with *Metassemble*r (Debruijn, 2014) using *Velvet* (Zerbino & Birney, 2008), run with different *k*-mer sizes ranging between 19 and 75, and were subsequently merged with *Minimus2* (Treangen et al., 2011). This assembly procedure resulted in a larger assembled dataset than assembly with *SOAPdenovo2* (Luo et al., 2012), *Metavelvet* (Namiki et al., 2012) or *Newbler* (Roche, 2014). Contig sequences longer than 1000 bp were considered further. The contigs were subsequently mapped with *BLAST* (Camacho et al., 2009) onto the reference genomes to recover their taxonomic identifiers.

Properties of the simulated datasets:

Distribution	Contigs	Mb
Uniform	14,393	137
Log-normal	13,284	66

List of strains used to generate simulated datasets:

Strain name	Accession number
Acidobacterium capsulatum ATCC 51196	CP001472.1
Akkermansia muciniphila ATCC BAA-835	CP001071.1
Archaeoglobus fulgidus DSM 4304	AE000782.1
Bacteroides thetaiotaomicron VPI-5482	AE015928.1
Bacteroides vulgatus ATCC 8482	CP000139.1
Bordetella bronchiseptica RB50	BX470250.1
Caldicellulosiruptor bescii DSM 6725	CP001393.1
Caldicellulosiruptor saccharolyticus DSM 8903	CP000679.1
Chlorobium limicola DSM 245	CP001097.1
Chlorobium phaeobacteroides DSM 266	CP000492.1
Chlorobium phaeovibrioides DSM 265	CP000607.1
Chlorobium tepidum TLS	AE006470.1
Chloroflexus aurantiacus J-10-fl	CP000909.1
Clostridium thermocellum ATCC 27405	CP000568.1
	AE001825.1
Deinococcus radiodurans R1	AE000513.1
Dickeya dadantii 3937	CP002038.1
Dictyoglomus turgidum DSM 6724	CP001251.1
Enterococcus faecalis V583	AE016830.1
Fusobacterium nucleatum subsp. *nucleatum* ATCC 25586	AE009951.2
Gemmatimonas aurantiaca T-27	AP009153.1
Herpetosiphon aurantiacus DSM 785	CP000875.1
Hydrogenobaculum sp. Y04AAS1	CP001130.1
Ignicoccus hospitalis KIN4/I	CP000816.1
Methanocaldococcus jannaschii DSM 2661	L77117.1
Methanococcus maripaludis C5	CP000609.1
Methanococcus maripaludis S2	BX950229.1
Nitrosomonas europaea ATCC 19718	AL954747.1
Pelodictyon phaeoclathratiforme BU-1	CP001110.1
Persephonella marina EX-H1	CP001230.1

Porphyromonas gingivalis ATCC 33277	AP009380.1
Pyrobaculum aerophilum str. IM2	AE009441.1
Pyrobaculum calidifontis JCM 11548	CP000561.1
Rhodopirellula baltica SH 1	BX119912.1
Ruegeria pomeroyi DSS-3	CP000031.1
Salinispora arenicola CNS-205	CP000850.1
Salinispora tropica CNB-440	CP000667.1
Shewanella baltica OS185	CP000753.1
Shewanella baltica OS223	CP001252.1
Sulfolobus tokodaii str. 7	BA000023.2
Sulfurihydrogenibium sp. YO3AOP1	CP001080.1
Thermoanaerobacter pseudethanolicus ATCC 33223	CP000924.1
Thermotoga neapolitana DSM 4359	CP000916.1
Thermotoga petrophila RKU-1	CP000702.1
Thermotoga sp. RQ2	CP000969.1
Thermus thermophilus HB8	AP008226.1
Treponema denticola ATCC 35405	AE017226.1
Zymomonas mobilis subsp. *mobilis* ZM4	AE008692.2

3.2 Real datasets

For the evaluation using real metagenome samples from actual microbial communities, we used two metagenome samples from the guts of obese human twins (Turnbaugh et al., 2010) and the dataset of a lignocellulose-degrading community from within a cow rumen (Hess et al., 2011).

3.2.1 Human gut dataset

The contigs from both samples, TS28 and TS29, were pooled. In the same way, scaffolds from TS28 and TS29 were pooled. All scaffolds were longer than 1000 bp. The dataset was generated with a 454 GS FLX Titanium sequencer.

Properties of the real human gut dataset:

FASTA file	Sequences	Mb
Contigs	153,564	255.2
Contigs ≥ 1000 bp	63,399	187.1
Scaffolds	18,172	164.4

3.2.2 Cow rumen dataset

The same dataset as in Dröge *et al.* (Dröge, Gregor & McHardy, 2014) was used. As the scaffolds of the assembled contigs were of lower quality than the contigs, scaffolds were split into contigs at all gaps consisting of at least 200 "N" characters. We subsequently split the resulting contigs of at least 10 kb into 'chunks' of 2000 bp, resulting in at least five chunks for each contig. The dataset was generated with Illumina GAIIx and Illumina HiSeq 2000 sequencers.

Properties of the real chunked cow rumen dataset:

FASTA file	Sequences	Mb
Contigs	159,263	318.5
Scaffolds	12,192	369.4

3.3 Reference data

The NCBI taxonomy (Federhen, 2011), downloaded on 11/22/2012, was used as the reference taxonomy. The following reference databases from the NCBI were pooled to generate our reference sequence (RS) collection: NCBI genomes (downloaded on 11/22/2012), NCBI draft bacterial genomes (downloaded on 11/22/2012), the NCBI human microbiome project (downloaded on 10/16/2012) and NCBI RefSeq (Sayers et al., 2008) microbial version 56. Subsequently, duplicate sequences were removed to make the RS collection non-redundant. This RS collection contained sequences for 841 different genera, 2543 different species and 4516 different strains. The total size of the RS collection was 16 Gb.

In the marker gene (MG) analysis, the following MG sequence collections and HMM profiles were used: For the 16S and 23S MG analysis, bacterial and archaeal reference sequences from the SILVA database (Pruesse et al., 2007) were retrieved (version 111, released on 7/27/2012). The corresponding taxonomic identifiers were mapped onto the NCBI taxonomy. The resulting collection contained 126,742 sequences for 39,201 different species (199 Mb in total).

For the 5S MG analysis, MG sequences were retrieved from NCBI on 2/8/2013 via Maglott *et al.* (Maglott et al., 2004); the collection contained 12,424 sequences for 1278 species (5.8 Mb in total).

In addition, reference sequences for the following 31 bacterial marker gene families were retrieved from NCBI on 2/8/2013 via Maglott *et al.* (Maglott et al., 2004): *dnaG*, *infC*, *pgk*, *rpoB*, *tsf*, *frr*, *nusA*, *pyrG*, *rpmA*, *smpB*, *rpsC*, *rpsI*, *rpsK*, *rpsS*, *rpsB*, *rpsE*, *rpsJ*, *rpsM*, *rplA*, *rplB*, *rplC*, *rplD*, *rplE*, *rplF*, *rplK*, *rplL*, *rplM*, *rplN*, *rplP*, *rplS* and *rplT*. This MG collection contained 63,530 sequences for 1380 different species (52 Mb in total).

HMM profiles for the 16S, 23S, and 5S marker genes were retrieved from Huang *et al.* (Huang, Gilna & Li, 2009) HMM profiles trained on the protein families for the 31 bacterial MG were retrieved from Wu & Scott. (Wu & Scott, 2012)

3.4 Test environments

The benchmarks were run on different hardware configurations. When measuring runtime, Hardware Configurations 1 or 2 were used if not stated otherwise.

1. Server: AMD Opteron Processor 6386 SE, 2.8 GHz; 512 GB RAM; local SSD storage; Debian GNU/Linux 7.1.
2. Laptop: Intel i5 M520 2.4 GHz; 4 GB RAM; 7200 rpm laptop storage; Windows 7 64-bit, Ubuntu 12.04 64-bit; Oracle VirtualBox 4.2.12: 2 GB RAM, 8 GB swap, 140 GB HDD, Ubuntu 12.04 64-bit.
3. Server: Intel Xeon CPU X5660, 2.8 GHz; 73 GB RAM; network storage; Debian GNU/Linux 6.0.7.
4. Server: AMD Opteron Processor 6174, 2.2 GHz; 100 GB RAM; local storage; Debian GNU/Linux 6.0.7.
5. Laptop: Intel i5 2557M 1.7 GHz; 4GB RAM, SSD storage, OS X 10.7.

3.5 *MEGAN4* configuration

NCBI BLAST (version 2.2.27+) was used to generate alignments (Section 3.4, HW Configuration 1), using 15 threads; the tabbed output format (7) was used. *MEGAN4* (4.70.4)

(Huson et al., 2011) was used for taxonomic assignment on a laptop (Section 3.4, HW Configuration 2) using the following settings: *minsupport=5, minscore=2, toppercent=20, mincomplexity=0.44*. The runtime of *MEGAN4* was just a few seconds, as the *LCA* algorithm it uses is simple and fast. Construction of the *BLAST* database from the reference sequence collection required 6 h 55 m, with the size of the database being 4 GB. To simulate the new strain, species and genus scenarios (Table 1: Test Scenarios 5, 8 and 9), the corresponding alignments of sequences present in both the test and reference data were removed from the *BLAST* output.

Runtimes of *BLAST* for the different metagenome datasets:

Dataset	Runtime
Simulated uniform	52 m 11 s
Simulated log-normal	19 m 18 s
Chunked cow rumen (contigs)	43 m 29 s
Chunked cow rumen (scaffolds)	42 m 56 s
Human gut (contigs)	44 m 05 s
Human gut (scaffolds)	25 m 37 s

3.6 *Taxator-tk* configuration

LAST (version 287) (Frith, Hamada & Horton, 2010) was used to produce alignments using one thread, output format 1 (maf). Constructing the *LAST* database for the reference sequence database required 81 h 29 min. The size of the resulting database was 91 Gb (Section 3.4, HW Configurations 1 and 4).

Taxator-tk (Dröge, Gregor & McHardy, 2014) was then employed to process metagenome sequence fragments using 15 threads and to produce taxonomic assignments using one thread for the input sequences (Section 3.4, HW Configuration 4). For the simulated datasets, the corresponding alignments of sequences present in both the test and reference data were removed to simulate the new strain, species and genus scenarios (Table 1: Test Scenarios 5, 8 and 9).

Commands

LAST command:

```
lastal -f 1 lastDb query.fna | lastmaf2alignments.py | sort | gzip > alignments.gz
```

BLAST command:

```
blastn -db blastDb -query query.fna -num_threads 15 -outfmt '6 qseqid qstart qend qlen sseqid sstart send bitscore evalue nident length' -out alignments.blast
```

Produce fragments:

```
cat alignments.blast | alignments-filter -b 50 | taxator -a rpa -q query.fna -f ref.fna -g ref_all.tax -p 15 | sort > fragments.gff3
```

Produce assignments:

```
cat fragments.gff3 | binner > assignments.tax
```

Runtimes of *LAST* for the different metagenome datasets:

Dataset	Runtime (HC 1)	Runtime (HC 4)
Simulated uniform	9 h 56 m 27 s	12 h 10 m 57 s
Simulated log-normal	5 h 02 m 03 s	6 h 16 m 02 s
Chunked cow rumen (contigs)	12 h 23 m 29 s	15 h 39 m 24 s
Chunked cow rumen (scaffolds)	15 h 15 m 20 s	19 h 15 m 12 s
Human gut (contigs)	10 h 29 m 12 s	13 h 48 m 57 s
Human gut (scaffolds)	7 h 41 m 05 s	10 h 16 m 20 s

Runtimes of *taxator-tk* for different metagenome datasets:

Dataset	Process fragments	Bin
Simulated uniform	36 h 54 m 02 s	17.4 s
Simulated uniform (new strain)	8 h 53 m 20 s	18.2 s
Simulated uniform (new species)	4 h 44 m 27 s	18.1 s
Simulated uniform (new genus)	54 m 39 s	17.5 s
Simulated log-normal	25 h 25 m 49 s	16.8 s
Simulated log-normal (new strain)	3 h 09 m 16 s	17.9 s
Simulated log-normal (new species)	2 h 06 m 29 s	17.4 s
Simulated log-normal (new genus)	36 m 34 s	16.9 s
Chunked cow rumen (contigs)	3 h 03 m 07 s	24.9 s
Chunked cow rumen (scaffolds)	46 m 59 s	19.2 s
Human gut (contigs)	6 h 38 m 56 s	22.5 s
Human gut (scaffolds)	2 h 47 m 50 s	18.6 s

3.7 *PPS*+ and *PPS* generic model configurations

PPS+ benchmarks were run using one thread (Section 3.4, HW Configuration 3). The *PPS*+ configuration file contained in the VM distribution specifies the default values of the parameters used (configuration file name: config_ppsp_vm_refNCBI20121122_example.cfg).

PPS was run using one thread (Section 3.4, HW Configuration 3). *PPS* was trained to include the 200 most abundant genera in the reference sequences (Section 3.3). The *PPS* models were built down to the genus rank, as this is the default setting of *PPS*.

3.8 *Kraken* configuration

Kraken (version 0.10.5) and its dependency *Jellyfish* (1.1.11) were installed on a high-performance server (Section 3.4, HW Configuration 1). Four *Kraken* databases were built using our custom reference data collection (Section 3.3). For the real datasets (Section 3.2) and the simulated datasets (Sections 3.1) – for the first scenario (Table 1: Test Scenarios 1), *kraken_db_all* database was built from all the reference sequence data (Section 3.3). To simulate the new strain, new species and new genus scenarios (Table 1: Test Scenarios 5, 8 and 9), we generated corresponding *Kraken* databases *kraken_db_new_strain*, *kraken_db_new_species* and *kraken_db_new_genus*. For instance, *kraken_db_new_strain* database does not contain the strains from which the simulated datasets were generated. When we use the *kraken_db_new_strain* database, we simulate the scenario in which all strains of a metagenome sample are unknown, i.e. (Table 1: Test Scenarios 5). This approach ensures that all the methods in comparison use the same reference data for the classification in respective test scenarios (Table 1). For instance, to create the *Kraken kraken_db_all* database, we performed the following steps:

1. Create directory *for_kraken_all* containing all the reference sequences that are used to build a custom reference database. Note that the sequence names in the FASTA files have to be in the format specified in the *Kraken* documentation.
2. Create empty directory *kraken_db_all* for the generated database.
3. Inside directory *kraken_db_all*, create directory *taxonomy* and place there the following NCBI taxonomy files: *gi_taxid_nucl.dmp, names.dmp, nodes.dmp.*
4. Switch to directory *for_kraken_all* and run the following command to add all the reference sequences to the *Kraken* database *kraken_db_all*:

*for file in *.fna; do kraken-build --add-to-library $file --db kraken_db_all --threads 40; done*

5. Set the *PATH* variable to contain also the installation *bin* directory of *Jellyfish*.
6. Build the *Kraken kraken_db_all* database:
   kraken-build --build --db kraken_db_all --threads 40
7. Perform taxonomic assignment of contigs contained in FASTA file *contigs.fna* and store the results in *contigs_lab.csv*:
   kraken --preload --db kraken_db_all --threads 40 contigs.fna > contigs_lab.csv

3.9 Assignment quality measures

3.9.1 Micro-averaged precision and recall

To assess the quality of the taxonomic assignments for the simulated datasets, we evaluated the micro-averaged precision (sometimes also known as the micro-averaged specificity) and the micro-averaged recall (sometimes also known as the micro-averaged sensitivity) of taxonomic assignments for the different methods, as detailed below. Both measures were calculated based on the number of assigned bp for each taxonomic rank, instead of per assigned fragment, as the correct assignment of larger sequence fragments is more beneficial for the retrieval of “draft genome” bins than for short fragments.

The micro-averaged precision was defined as:

$$p^l = \frac{\sum_{i=1}^{N_p^l} TP_i^l}{\sum_{i=1}^{N_p^l} TP_i^l + FP_i^l};$$

and micro-averaged recall was defined as:

$$r^l = \frac{\sum_{i=1}^{N_r^l} TP_i^l}{\sum_{i=1}^{N_r^l} TP_i^l + FN_i^l},$$

where l denotes the taxonomic rank evaluated, such as species, genus, family, order, class, phylum or superkingdom; $(TP_i^l + FN_i^l)$ is the number of bp from taxon i; $(TP_i^l + FP_i^l)$ is the number of bp assigned to taxon i and TP_i^l is the number of bp correctly assigned to taxon i. The precision is micro-averaged over all bins N_p^l to which a sequence fragment was assigned and the recall is micro-averaged over all N_r^l taxa present in the simulated dataset at rank l.

The micro-averaged precision is the fraction of correctly assigned bp from all predictions for a particular taxonomic rank and represents a measure of confidence for the predictions of a method. The micro-averaged recall is the fraction of correct assignments of the test sample for a particular taxonomic rank. To avoid an uninformative increase of the micro-averaged recall by having unassigned sequences, which belong to no taxon at a given rank, our test datasets were generated from sequenced isolates with taxa defined at all major taxonomic ranks. Note that for simplification, we denoted the micro-averaged precision as 'precision' and the micro-averaged recall as 'recall' in this document.

3.9.2 Taxonomic assignment correction for assessment of bin quality

Often, a species within a metagenome sample is not directly represented among the reference sequences; however, this respective species is closely related to a species for which there is enough data in the RS or MG collections. In this case, the species from the sample may be consistently assigned to the closely related species. This error does not impact draft genome reconstruction in terms of reconstructing a bin as a set of sequences originating from the same sample population, but the assigned identifier itself is incorrect. To quantify the binning performance independently from taxonomic label assignment, we applied a correction procedure and re-computed the corrected precision and recall values: If most of the sequences (i.e. at least *(correctLabelThreshold * 100)% bp*) from one taxon were consistently assigned to a false identifier, their identifiers were changed to the correct one, and precision and recall were re-computed. The default setting for the configuration parameter *correctLabelThreshold* was 0.9. The precision and recall were always calculated with and without this correction.

3.10 Scaffold-contig consistency definitions

3.10.1 Comparison of scaffold and contig assignments

To assess the consistency of scaffold and contig assignments for a metagenome sample, we define the following measures at all major taxonomic ranks (i.e. superkingdom, phylum, class, order, family, genus and species). The idea of these measures is that each contig is assigned up to two taxonomic identifiers: one from the contig assignment and the other from the scaffold assignment. These two taxonomic labels are then compared. If we considered contigs with two identical taxonomic labels to be correctly assigned and contigs with two

distinct taxonomic labels to be as incorrectly assigned, then "% agreement" resembles a measure of precision (i.e. correctly assigned bp ÷ correctly and incorrectly assigned bp), while "kb agreement" indicates recall (i.e. the total number of correctly assigned bp).

Let us assume that a metagenome sample consists of m scaffolds $s_0, \ldots, s_{m-1}$ and n contigs $c_0, \ldots, c_{n-1}$, where scaffold s_k consists of n_k contigs $c_{k(0)}, \ldots, c_{k(n_k - 1)}$. Let function l denotes the taxonomic identifier of a contig or a scaffold at the taxonomic rank being considered, i.e. $l(c_i)$ is a label of the ith contig and $l(s_k)$ is the label of the kth scaffold. The lengths of contig c_i and scaffold s_k are denoted by $len(c_i)$ and $len(s_k)$, respectively. Now, we can define the consistency measures 'kb agreement' (Def. 0a) and '% agreement' (Def. 0b) as:

0a) 'kb agreement':

$$a_{kb} = \sum_{k=0}^{m-1} \sum_{j \in \{k(0),\ldots,k(n_k-1)\},\ l(s_k)\ and\ l(c_j)\ defined,\ l(s_k)=l(c_j)} len(c_j);$$

0b) '% agreement':

$$a_{\%} = \frac{a_{kb}}{\sum_{j=0}^{n-1} len(c_j)}.$$

In other words, in 'kb agreement' (Def. 0a), the index k goes over all scaffolds, the index j goes over all contigs within a corresponding scaffold. If both labels of scaffold k and contig j are defined and assigned to the same taxa, then the length of contig j is added to the overall sum of lengths of consistently assigned contigs.

3.10.2 Taxonomic scaffold-contig assignment consistency

To provide more detailed insights into the evaluation of the binning results of real metagenome datasets, we introduced new detailed measures of the scaffold-contig consistency (described below).

We assume that all contigs $c_0,\ldots,c_{n-1}$ of a particular scaffold originated from the same organism and thus should be assigned the same taxonomic identifier. Let us denote an identifier of contig c_i as l_i. Each path p_i from the root of the taxonomy to identifier l_i represents a hypothesis about the identifier of the whole scaffold. We base our definition on the assumption that the most representative identifier of a scaffold corresponds to the path to which the identifiers of all taxonomically assigned contigs that do not lie on the path have the

shortest collective weighted distance. Note that we do not have to consider the path p_i from the root to l_i as a potential taxonomic identifier if there is a path p_j from the root to the taxonomic identifier l_j of another contig c_j for which l_i lies on p_j and $i \neq j$, as the shortest collective weighted distance of all contigs of a scaffold to path p_j is always lower than the collective weighted distance to path p_i. Let us denote the length of contig c_i as $|c_i|$ (counted in bp). Let us define the weight of contig c_i as $w_i = \frac{|c_i|}{\sum_{j=0}^{n-1}|c_j|}$. Let $tax\_dist(l_i, p_j)$ be the taxonomic distance (i.e. the number of edges in the reference taxonomy) between identifier l_i and the closest identifier l_k that lies on path p_j (i.e. this is simply the distance between identifier l_k and path p_j). The weighted distance from path p_j to all other identifiers l_i is defined as: $dist(p_j) = \sum_{i=0}^{n-1} w_i * tax\_dist(l_i, p_j)$. Let p_k be the path with the minimum weighted distance (*dist*) from all other identifiers. All contigs c_i that lie on path p_k are considered to be consistently assigned within the scaffold; all contigs c_j that do not lie on the path are considered to be inconsistent. The consistency of the scaffold is then defined as:

1) Proportion of consistently assigned contigs:

$$\frac{|\{c_i \mid l_i \ on \ p_k\}|}{|\{c_i \mid i=0 \ldots n-1\}|};$$

2) Proportion of consistent contigs in bp:

$$\frac{\sum_{\{i \mid l_i \ on \ p_k\}} |c_i|}{\sum_{i=0}^{n-1} |c_i|};$$

3) Average distance to the path:

$$\frac{\sum_{i=0}^{n-1} tax\_dist(l_i, \ p_k)}{n};$$

4) Average weighted distance to the path:

$$dist(p_k);$$

5) Average distance to the scaffold identifier:

$$\frac{\sum_{i=0}^{n-1} tax\_dist(l_i, \ l_k)}{n};$$

6) Average weighted distance to the scaffold identifier:

$$\sum_{i=0}^{n-1} w_i * tax\_dist(l_i, l_k).$$

The first definition is the coarsest measure and the last is the finest for taxonomic assignment consistency.

We can also group the scaffolds using l_k and compute the measures for individual taxa. However, these groups do not correspond to the assigned bins, as a scaffold's taxonomic identifier does not always correspond to the taxonomic identifier of the lowest assigned contig of that scaffold.

The consistency of the entire sample can also be defined as the (weighted) average of these measures. Let $s_0, \ldots, s_{m-1}$ be all scaffolds in the sample, where if a contig is not assigned to a scaffold, an artificial scaffold that contains this one contig is created. We can also consider only scaffolds that contain only a certain number of contigs or those that are at least x bp long, for example.

Thus if we compute these measures for two different binning methods, we can assess the consistency of the respective taxonomic assignments at six different levels. However, be aware that it is recommended to also look at the number of bp assigned at different taxonomic ranks by each method, since the consistency of a method that assigns everything to the root of the taxonomy seems to be perfect according to these scaffold-contig consistency definitions.

4 Detailed results for the simulated datasets

This section provides a detailed description of the results of the benchmarks with simulated datasets in nine different test scenarios (Table 1). *PPS*+, *PPS* generic model, *MEGAN4* and *taxator-tk* were compared to each other in terms of precision and recall (Section 3.9). The nine different scenarios evaluate assignment performances for different evolutionary distances between the sample sequences and the available reference sequences. For instance, in (Table 1: Test Scenario 6), all sequences from the species included in the simulated communities were excluded from the reference sequence collection and all sequences of the same strains were excluded from the marker gene sequence collection.

4.1 Uniform dataset

For *PPS*+, a drop in both precision and recall was only observed for low-level taxonomic assignments when removing reference data from the same strain, species or genera from the reference sequence (RS) collection and also from the MG collection (Table 1: Test Scenarios 2, 3 and 4 versus Test Scenarios 5, 8 and 9), which demonstrated that for microbial community members that have been profiled by 16S sequencing but which have no sequenced genomes available, *PPS*+ can perform highly accurate low-level taxonomic assignments, unlike from all other tested methods (Figs S1a and S1c–S1f).

In more detail, *PPS*+ showed substantially higher precision and recall than the *PPS* generic model for all test scenarios (Fig. S1a–S1d, Table 1: Test Scenarios 1–9). *PPS*+ also showed substantially higher precision and recall than *MEGAN4* for the assignment of sequences from new strains, species and genera (Figs S1a and S1e, Table 1: Test Scenarios 2–4), when these were represented in the reference collection as marker genes. An exception was the unrealistic case, when all of the simulated metagenome data were available in the reference sequence collection (Table 1: Test Scenario 1).

Simulating the situation where the microbial community members have not been observed in profiling before, we removed these strains from the MG collection and the reference sequences (RS) for the strains, species or genera of the simulated metagenome datasets (Table 1: Test Scenarios 5, 6 and 7). We removed more data from the reference sequence (RS) collection than from the MG collection to simulate the situation where a closer relative can be found among the marker genes and a more distant one among the sequenced genomes, as many taxa have been profiled but have not had their genomes sequenced. *PPS*+ assignment quality (both precision and recall) dropped in comparison to the situation where strains have been profiled (Fig. S1a,b). However, it was still better than *MEGAN4* (Figure S1e) for all ranks, except for the lowest-level assignment (species), when the strains were removed from the RS collection only (Table 1: Test Scenario 5). As the removal of strain-level data in many cases also removed all data for the respective species from the RS collection, both methods made false assignments to related species in these scenarios.

When we removed even more reference data from the MG collection to simulate the binning of microbial community members for which no members of the same species or genera have

been profiled or sequenced before (Figure S1c, Table 1: Test Scenarios 8 and 9), the precision for ranks above remained high (Table 1: Test Scenario 8, genus rank: 88.5%; Test Scenario 9, family rank: 73.2%), while the recall dropped moderately. However, *PPS+*'s assignments were still substantially better than those of *MEGAN4* for these ranks (Figure S1e, Test Scenario 8, genus rank: 81.6%; Test Scenario 9, family rank: 58.9%). For lower ranks for which all reference data were removed, both methods had low precision and recall due to false positive assignments.

Taxator-tk showed a lower recall than *PPS+* across all tested scenarios (Figs S1a–S1c and S1f), but showed outstanding precision for the order rank and above (close to 100%), and never dropped below 89% at lower ranks. Thus this method could also be used for taxonomic profiling to determine the presence of particular taxa reliably in a given sample.

4.2 Log-normal dataset

Even though the log-normal dataset was more challenging for all the tools, this benchmark yielded similar conclusions as the benchmark with the uniform dataset.

PPS+ performed substantially better than the generic *PPS* model in terms of the precision and recall in all test scenarios (Fig. S3a–S3d, Table 1: Test Scenarios 1–9).

At low taxonomic ranks (i.e. family, genus and species), *PPS+* outperformed *MEGAN4* in terms of precision and recall in almost all test scenarios (Figs S3a–S3c and S3e, Table 1: Test Scenarios 2–9), except at the family rank in the 'new strain' scenario, where *MEGAN4* had slightly better precision (96.7%) than *PPS+* (94.8%) (Figs S3b, S3c and S3e, Table 1: Test Scenario 5). In the unrealistic case, where all reference data remained in the reference (RS and MG) collections, *MEGAN4* had better precision and recall (Figs S3a–S3c and S3e, Table 1: Test Scenario 1).

Overall, *PPS+* showed substantially better recall than *taxator-tk*, whereas *taxator-tk* showed mostly better precision (Figs S3a–S3c and S3f, Table 1: Test Scenarios 1–9). Moreover, in the case where microbial community members have been profiled by 16S but have no sequenced genomes, *PPS+* showed a very high precision at low taxonomic ranks (i.e. family, genus and species) 99.5–89.6% (Figs S3a and S3f, Table 1: Test Scenarios 2–4). In several cases, *PPS+*

showed better precision than *taxator-tk*; for example, at the family rank, the precision was 98.1% for *PPS*+ vs 91.9% for *taxator-tk* (Figs S3a and S3f, Table 1: Test Scenario 4) and at the genus rank, it was (scenario 2) 96.1%, (scenario 3) 96.3% for *PPS*+ vs (scenario 2) 91%, (scenario 3) 86.9% for *taxator-tk* (Figs S3a and S3f, Table 1: Test Scenarios 2, 3).

4.3 Benchmarks with corrections

In the test scenarios when the reference data were excluded from the MG database (Table 1: Test Scenarios 5–9), the precision of *PPS*+ for low taxonomic ranks (i.e. genus and species) was lower than the precision of *taxator-tk* because of the way *PPS*+ chooses the taxa that are modeled. If the sequences from the same strains as those of the simulated metagenome samples were removed from the MG reference database at the strain, species or genus ranks, the marker gene analysis assigned sequences of the metagenome sample that would otherwise have a very good match to the respective MG database sequences to corresponding closely related taxa.

In the subsequent *PPS* training phase, the sample-derived data were used to train closely related clades; moreover, reference sequences from closely related clades were used as training data as well. However, for the draft genome reconstruction, it is necessary to infer consistent bins from a metagenome sample. The actual identifiers of the bins are of lower importance. Therefore, we recomputed the precision and recall measures with a correction to account for the phenomenon described above (Section 3.9, Figs S2a–S2f and S4a–S4f, Table 1: Test Scenarios 1–9).

The corrected precision of *PPS*+ was substantially better than it was without the correction for all scenarios. The difference for the other methods is not that pronounced, since they choose clades to which metagenome sequences are assigned in a different way. When comparing *PPS*+ to *MEGAN4* using these corrections, *PPS*+ showed higher precision and recall. When comparing *PPS*+ to *taxator-tk*, *PPS*+ had higher recall; however, neither method was consistently more precise.

5 Detailed results for the real datasets

5.1 Taxonomic scaffold-contig assignment consistency

To assess the quality of taxonomic assignments for these samples, we evaluated the consistency of taxonomic assignments for contigs originating from the same scaffold using a set of measures (Section 3.10.2). These measures assessed the degree to which the taxonomic identifiers of scaffolds and their constituent contigs were consistent relative to each other. This method looked beyond identical identifiers (Section 3.10.1) by taking the relative distances between two taxa in the reference taxonomy into account (Table S6 and S7).

The basic idea of these measures is that a scaffold is assigned to a taxonomic identifier of one of its constituent contigs, such that the collective distance of all contig assignments for the respective scaffold to path *p* in the taxonomy defined by the scaffold identifier is the shortest. The consistency of individual contig assignments is then assessed relative to path *p*: If a contig lies on *p*, it is considered to be assigned consistently; if it does not lie on *p*, it is assigned inconsistently. These measures were computed for the assignments of the chunked cow rumen and the human gut datasets.

Overall, *PPS+* performed better in terms of the consistent assignment of sequences to low taxonomic ranks for the chunked cow rumen dataset and the human gut dataset than the generic *PPS* model and *MEGAN4* (Table S6 and S7, Def. 6). For both datasets, *taxator-tk* showed the highest consistency according to almost all measures; however, it assigned fewer data to lower taxonomic ranks (family, genus and species) than the other methods.

For the chunked cow rumen dataset, the generic *PPS* model assigned more contigs consistently than *PPS+* (Table S6, Def. 2); however this came at the cost of many contigs being assigned to higher taxonomic ranks by *PPS* (Table S6, Defs 0a, 6). *MEGAN4* showed a higher overall consistency than *PPS+* (Table S6, Def. 2) but this was mostly due to many contigs being assigned at higher taxonomic ranks (Table S6, Def. 6). For lower taxonomic ranks or when also taking sequence length into account (instead of the number of assigned sequences), *MEGAN4* was less consistent than *PPS+* (Table S6, Defs 0b, 3–6).

For the human gut dataset, *PPS*+ performed better than the generic *PPS* model according to all measures (Table S7, Def. 0–6). *PPS*+ was again more consistent than *MEGAN4* when taking sequence lengths into account (Table S7, Defs 2, 4, 6). These measures are more informative for taxonomic binning than the sequence-count based measures (Table S7, Defs 1, 3, 5), as obtaining large bins is desirable. These results also imply that *MEGAN4* assigned substantially more (predominantly short) sequences to lower taxonomic ranks than *PPS*+ (Table S7, Def. 0a).

5.2 Evaluation summary

Our evaluation showed that *PPS*+ performed substantially better than the generic *PPS* model (Tables 2–3, S6–S7). Moreover, the results of *PPS*+ were comparable to a sample-derived model generated according to expert specifications (Table 4). *Taxator-tk* had the highest consistency of all the methods; however, it assigned substantially fewer sequences to low taxonomic ranks than the other methods (Tables 2–3, S6–S7). Our benchmark experiments also confirmed that if the metagenome sequences were closely related to the reference sequences, such as for the human gut dataset, the homology-based methods assigned more sequences correctly to low taxonomic ranks than they did across larger taxonomic distances, as was the case for the cow rumen dataset (Tables 2–3, S6–S7). *PPS*+ was not that sensitive to this distance. For *PPS*+, only few taxonomically informative marker genes have to be identified from the sample, for which a substantially larger marker gene reference collection exists than that for genome and draft genome sequences, in terms of the number of species represented in the reference collection. *PPS*+ often made more consistent assignments than *MEGAN4* and often assigned the most sequences of all the tested methods to lower taxonomic ranks (Tables 2–3, S6–S7).

5.3 Throughput comparison

The throughput of the individual methods for contig assignments of the human gut sample was calculated as either Mb or the number of sequences assigned per hour with one thread using the same reference sequences (Sections 3.3 and 3.4). *PPS* and *PPS*+ directly use sequences in FASTA format as references, while for *MEGAN4* and *taxator-tk BLAST* or *LAST* databases were initially constructed. Database construction took 6 h 55 m and 81 h 29 min on

our servers, respectively for *BLAST* and *LAST*, and was not considered in runtime comparisons. As most time in *PPS*+ is spent with model construction, assignment can be further accelerated when reusing models to classify multiple metagenome samples. In this setting, where we consider only the prediction phase of *PPS*+, *PPS*+ was more than 7 times faster (up to 0.5 Gb/h) than the homology-based methods (Figure 4). As only a relatively small reference sequence database of 16 Gb was used, runtimes of *BLAST* and *LAST* searches in the homology-based tools would proportionally increase when using larger reference collections.

Unlike the homology-based tools, for which similarity searches require the use of more hardware with more CPUs and main memory, *PPS*+ can run on a standard laptop computer. *PPS*+ on a laptop with an Intel i5 M520 2.4 GHz processor and 4 GB of RAM was ~1.5–4 times slower than it was on the server with an AMD Opteron 6386 SE 2.8 GHz processor and 512 GB of RAM, mainly due to insufficient RAM on the laptop installed, which caused extensive use of the swap space.

6 External tools

6.1 HMMER 3

The search command (*hmmsearch*) of the *HMMER 3* (Eddy, 2011) package with e-value cut-off set to 0.01 is used.

6.2 MOTHUR

The *MOTHUR* (Schloss et al., 2009) Naïve Bayes classifier with the following default parameters is used. The number of bootstrap replicas is set to 300. The corresponding confidence score cut-off is set to 80. For the 16S analysis (i.e. 3 (5S, 16S, 23S) out of 34 marker genes), a small part of the code from (Huang, Gilna & Li, 2009) was used.

7 Evaluation of the *CLARK* software

CLARK (Ounit et al., 2015) is a straightforward, fast, taxonomy-free, *k*-mer based binning tool for metagenome reads and contigs. It is a taxonomy-free tool, since a user has to first

decide, on which taxonomic rank s/he would like to assign sequences of a metagenome sample, and then a taxonomic identifier is assigned to all input sequences at a particular taxonomic rank. However, this is different from taxonomic binning tools such as *PPS+*, *taxator-tk* (Dröge, Gregor & McHardy, 2014), *MEGAN* (Huson et al., 2011), or *Kraken* (Wood & Salzberg, 2014), since a taxonomic binning tool first has to automatically decide on a taxonomic rank on which a sequence will be assigned and then it assigns a taxonomic identifier to the sequence at a particular rank. In *CLARK*, the first step has to be done manually, which makes the tool unsuitable for the analysis of metagenome samples originating from novel environments. For instance, if a metagenome sample contained species, that were all novel species and a user decided to assign all the sequences of the sample at the species rank, then all the assignments would have been incorrect. Therefore, it is an indispensable feature of a taxonomic metagenome binning tool to also automatically and correctly determine a taxonomic rank of an assignment. This makes the application of *CLARK* limited only to the environments that has been well studied, for which there have been many reference (draft) genomes sequenced, and that does not contain novel taxa. For such, well studied, environments, *CLARK* offers a substantial speed-up in comparison to, e.g. *BLAST* (Camacho et al., 2009). Nevertheless, it is unsuitable for the analysis of metagenome samples originating from novel environments.

We have evaluated *CLARK* in the "new strain", "new species", and "new genus" scenarios with a simulated dataset with uniform distribution (Section 3.1). For the "new strain" scenario, we have excluded all the strains of the simulated dataset from the reference sequence collection and built the *CLARK* reference database at the species rank. In this "new strain" scenario, the precision of *CLARK* at the species rank was 36.8% and recall 24.7%. The corrected measures were 57.3% and 37.6%, respectively (Section 3.9).

For the "new species" scenario, we have excluded all the species of the simulated dataset from the reference sequence collection and built the *CLARK* reference database at the genus rank. In this "new species" scenario, the precision at the genus rank was 83.2% and recall 57.9%. The corrected measures were 85.1% and 59.6%, respectively.

For the "new genus" scenario, we have built the *CLARK* reference database at the family rank. In this "new genus" scenario, the precision at the family rank was 57.3% and recall 33.3%. The corrected measures were 57.6% and 33.8%, respectively.

Note that these precision and recall values cannot be directly compared to the results of other taxonomic binning methods, as we have manually determined, on which taxonomic rank the

assignments were made by building the *CLARK* reference database at a particular rank. However, if the *CLARK* was extended from taxonomy-free binning software to a taxonomy binning software, its performance would be similar to *Kraken*, as both methods are based on the occurrences of long *k*-mers (k≈31).

8 References

Albertsen M, Hugenholtz P, Skarshewski A, Nielsen KL, Tyson GW, Nielsen PH 2013. Genome sequences of rare, uncultured bacteria obtained by differential coverage binning of multiple metagenomes. *Nature Biotechnology* 31:533–538.

Audano P, Vannberg F 2014. KAnalyze: a fast versatile pipelined K-mer toolkit. *Bioinformatics* 30:2070–2072.

Camacho C, Coulouris G, Avagyan V, Ma N, Papadopoulos J, Bealer K, Madden TL 2009. BLAST+: architecture and applications. *BMC bioinformatics* 10:421–421.

Debruijn I 2014. MetAssemble. *Available at https://github.com/inodb/metassemble/* (accessed January 23, 2014).

Dröge J, Gregor I, McHardy AC 2014. Taxator-tk: Fast and Reliable Taxonomic Assignment of Metagenomes by Approximating Evolutionary Neighborhoods. *Bioinformatics*.

Eddy SR 2011. Accelerated Profile HMM Searches. *PLoS Computational Biology* 7:e1002195–e1002195.

Federhen S 2011. The NCBI Taxonomy database. *Nucleic Acids Research* 40:D136–D143.

Frith MC, Hamada M, Horton P 2010. Parameters for accurate genome alignment. *BMC Bioinformatics* 11:80.

Hess M, Sczyrba A, Egan R, Kim T-W, Chokhawala H, Schroth G, Luo S, Clark DS, Chen F, Zhang T, Mackie RI, Pennacchio LA, Tringe SG, Visel A, Woyke T, Wang Z, Rubin EM 2011. Metagenomic discovery of biomass-degrading genes and genomes from cow rumen. *Science* 331:463–467.

Hu X, Yuan J, Shi Y, Lu J, Liu B, Li Z, Chen Y, Mu D, Zhang H, Li N, Yue Z, Bai F, Li H, Fan W 2012. pIRS: Profile-based Illumina pair-end reads simulator. *Bioinformatics* 28:1533–1535.

Huang W, Li L, Myers JR, Marth GT 2012. ART: a next-generation sequencing read

simulator. *Bioinformatics* 28:593–594.

Huang Y, Gilna P, Li W 2009. Identification of ribosomal RNA genes in metagenomic fragments. *Bioinformatics* 25:1338–1340.

Huson DH, Mitra S, Ruscheweyh HJ, Weber N, Schuster SC 2011. Integrative analysis of environmental sequences using MEGAN4. *Genome Research* 21:1552–1560.

Imelfort M, Parks D, Ben J Woodcroft, Dennis P, Hugenholtz P, Tyson GW 2014. GroopM: An automated tool for the recovery of population genomes from related metagenomes. *PeerJ*.

Iverson V, Morris RM, Frazar CD, Berthiaume CT, Morales RL, Armbrust EV 2012. Untangling Genomes from Metagenomes: Revealing an Uncultured Class of Marine Euryarchaeota. *Science* 335:587–590.

Luo R, Liu B, Xie Y, Li Z, Huang W, Yuan J, He G, Chen Y, Pan Q, Liu Y, Tang J, Wu G, Zhang H, Shi Y, Liu Y, Yu C, Wang B, Lu Y, Han C, Cheung DW, Yiu S-M, Peng S, Xiaoqian Z, Liu G, Liao X, Li Y, Yang H, Wang J, Lam TW, Wang J 2012. SOAPdenovo2: an empirically improved memory-efficient short-read de novo assembler. *GigaScience* 1:1–6.

Maglott DD, Ostell JJ, Pruitt KDK, Tatusova TT 2004. Entrez Gene: gene-centered information at NCBI. *Nucleic Acids Research* 33:D54–D58.

Marcais G, Kingsford C 2011. A fast, lock-free approach for efficient parallel counting of occurrences of k-mers. *Bioinformatics* 27:764–770.

Mavromatis K, Ivanova N, Barry K, Shapiro H, Goltsman E, McHardy AC, Rigoutsos I, Salamov A, Korzeniewski F, Land M, Lapidus A, Grigoriev I, Richardson P, Hugenholtz P, Kyrpides NC 2007. Use of simulated data sets to evaluate the fidelity of metagenomic processing methods. *Nature Methods* 4:495–500.

McHardy AC, Martín HG, Tsirigos A, Hugenholtz P, Rigoutsos I 2007. Accurate phylogenetic classification of variable-length DNA fragments. *Nature Methods* 4:63–72.

Namiki T, Hachiya T, Tanaka H, Sakakibara Y 2012. MetaVelvet: an extension of Velvet assembler to de novo metagenome assembly from short sequence reads. *Nucleic Acids Research* 1–12.

Ounit R, Wanamaker S, Close TJ, Lonardi S 2015. CLARK: fast and accurate classification of metagenomic and genomic sequences using discriminative k-mers. *BMC Genomics* 16:236.

Patil KR, Haider P, Pope PB, Turnbaugh PJ, Morrison M, Scheffer T, McHardy AC 2011. Taxonomic metagenome sequence assignment with structured output models. *Nature*

Methods 8:191–192.

Pope PB, Smith W, Denman SE, Tringe SG, Barry K, Hugenholtz P, McSweeney CS, McHardy AC, Morrison M 2011. Isolation of Succinivibrionaceae implicated in low methane emissions from Tammar wallabies. *Science* 333:646–648.

Pruesse E, Quast C, Knittel K, Fuchs BM, Ludwig W, Peplies J, Glöckner FO 2007. SILVA: a comprehensive online resource for quality checked and aligned ribosomal RNA sequence data compatible with ARB. *Nucleic Acids Research* 35:7188–7196.

Richter DC, Ott F, Auch AF, Schmid R, Huson DH 2007. MetaSim: a sequencing simulator for genomics and metagenomics. *CORD Conference Proceedings* 3:e3373–e3373.

Roche 2014.Newbler. *Available at http://www.454.com/products/analysis-software/* (accessed January 23, 2014).

Sayers EW, Barrett T, Benson DA, Bryant SH, Canese K, Chetvernin V, Church DM, DiCuccio M, Edgar R, Federhen S, Feolo M, Geer LY, Helmberg W, Kapustin Y, Landsman D, Lipman DJ, Madden TL, Maglott DR, Miller V, Mizrachi I, Ostell J, Pruitt KD, Schuler GD, Sequeira E, Sherry ST, Shumway M, Sirotkin K, Souvorov A, Starchenko G, Tatusova TA, Wagner L, Yaschenko E, Ye J 2008. Database resources of the National Center for Biotechnology Information. *Nucleic Acids Research* 37:D5–15.

Schloss PD, Westcott SL, Ryabin T, Hall JR, Hartmann M, Hollister EB, Lesniewski RA, Oakley BB, Parks DH, Robinson CJ, Sahl JW, Stres B, Thallinger GG, Van Horn DJ, Weber CF 2009. Introducing mothur: open-source, platform-independent, community-supported software for describing and comparing microbial communities. *Applied and Environmental Microbiology* 75:7537–7541.

Treangen TJ, Sommer DD, Angly FE, Koren S, Pop M 2011. Next generation sequence assembly with AMOS. *Current Protocols in Bioinformatics* 11:11.8.1–11.8.18.

Turnbaugh PJ, Quince C, Faith JJ, McHardy AC, Yatsunenko T, Niazi F, Affourtit J, Egholm M, Henrissat B, Knight R, Gordon JI 2010. Organismal, genetic, and transcriptional variation in the deeply sequenced gut microbiomes of identical twins. *Proceedings of the National Academy of Sciences of the United States of America* 107:7503–7508.

Wood DE, Salzberg SL 2014. Kraken: ultrafast metagenomic sequence classification using exact alignments. *Genome Biology* 15:R46.

Wu M, Scott AJ 2012. Phylogenomic analysis of bacterial and archaeal sequences with AMPHORA2. *Bioinformatics* 28:1033–1034.

Zerbino DR, Birney EE 2008. Velvet: algorithms for de novo short read assembly using de Bruijn graphs. *Genome Research* 18:821–829.
