# Supplementary material for: PhyloPythiaS+: a self-training method for the rapid reconstruction of low-ranking taxonomic bins from metagenomes
Source: PeerJ. 2016 Feb 8;4:e1603. doi: 10.7717/peerj.1603 (PMC4748697; doi:10.7717/peerj.1603)
Supplement: Text S2 — Personal communication from Dr PB Pope. [file peerj-04-1603-s002.pdf]

Supplemental Text S2

PhyloPythiaS+: A Self-Training Method for the Rapid Reconstruction of Low-Ranking Taxonomic Bins from Metagenomes

Personal communication from Dr. P. B. Pope.

Norges miljø- og biovitenskapelige universitet

DEPARTMENT OF CHEMISTRY,
BIOTECHNOLOGY AND FOOD SCIENCE

P.O. BOX 5003
N-1432 ÅS

TELEPHONE: +47 6494 6232
E-MAIL: phil.pope@nmbu.no
E-MAIL: phillip.b.pope@gmail.com

October 7, 2014

2810 2005 To Whom It May Concern:

A PPS+ binning of shotgun metagenome samples indicated the likely metabolite flow and participating microbial phylotypes for a biogas-producing microbial community tolerant of high ammonia levels

Methane is the energy-rich component of biogas and is formed as the end product during anaerobic degradation of organic material in bioreactors by a consortium of mainly uncultured microorganisms. One of the key problems in biogas reactors are high ammonia levels, which are associated with unstable process performance and increased risk of process failure. Therefore, characterizing the microbiome structure and function within a stable biogas reactor operating at high ammonia levels (run on slaughterhouse and industrial lignocellulosic waste: SwRI-ha) was of considerable interest to us. From two replicate reactor samples we generated approximately 48 Gb of shotgun sequence using paired-end Illumina HiSeq sequencing and assembled these with SOAPdenovo. **PPS+ was then applied for taxon-bin recovery, which reconstructed and taxonomically assigned eight draft genomes bins (Table 1)**, including uncultured phylotypes of species representing syntrophic acetate-oxidizing bacteria, methanogens (non-acetoclastic) and different fermentative bacteria (carbohydrate and amino-acid). These bins thus likely represent organisms known to produce acetate from the reactor substrate, organisms known to convert the acetate to carbon dioxide and hydrogen gas, as well as for organisms producing methane from carbon dioxide and hydrogen gas as opposed to acetoclastic methanogens. A functional analysis of these bins revealed some of the essential genes for each of these pathways, in support of their putative roles. Thus, the taxonomic bins reconstructed with PPS+ from the shotgun metagenome samples allowed us to determine the likely metabolite flow from the substrates to the end product for a unique biogas-producing microbial community tolerant of high ammonia levels (Frank and Pope, personal communication).

Sincerely,

Phillip B. Pope

Department of Chemistry, Biotechnology and Food Science
Norwegian University of Life Sciences
Post Office Box 5003
1432, Ås
Norway
Phone: +47 6496 6232
Email: phil.pope@nmbu.no

Norges miljø- og biovitenskapelige universitet

TABLE 1

2810
2005

Genome ID	Base Pairs	Contigs	TaxonId	Scientific Name
pTaa-1	1358596	377	499229	Tepidanaerobacter acetatoxydans
pBah-1	910935	143	86665	Bacillus halodurans
pMcb-1	534996	177	83986	Methanoculleus bourgensis
pSmw-1	487779	158	863	Syntrophomonas wolfei
pMsb-1	292500	131	2208	Methanosarcina barkeri
pMml-1	292331	96	1080712	Methanomassiliicoccus luminyensis
pMcm-1	99651	53	2198	Methanoculleus marisnigri
pAbc-1	90549	53	81468	Aminobacterium colombiense
