# Supplementary figures and images for: PhyloPythiaS+: a self-training method for the rapid reconstruction of low-ranking taxonomic bins from metagenomes
Source: PeerJ. 2016 Feb 8;4:e1603. doi: 10.7717/peerj.1603 (PMC4748697; doi:10.7717/peerj.1603)

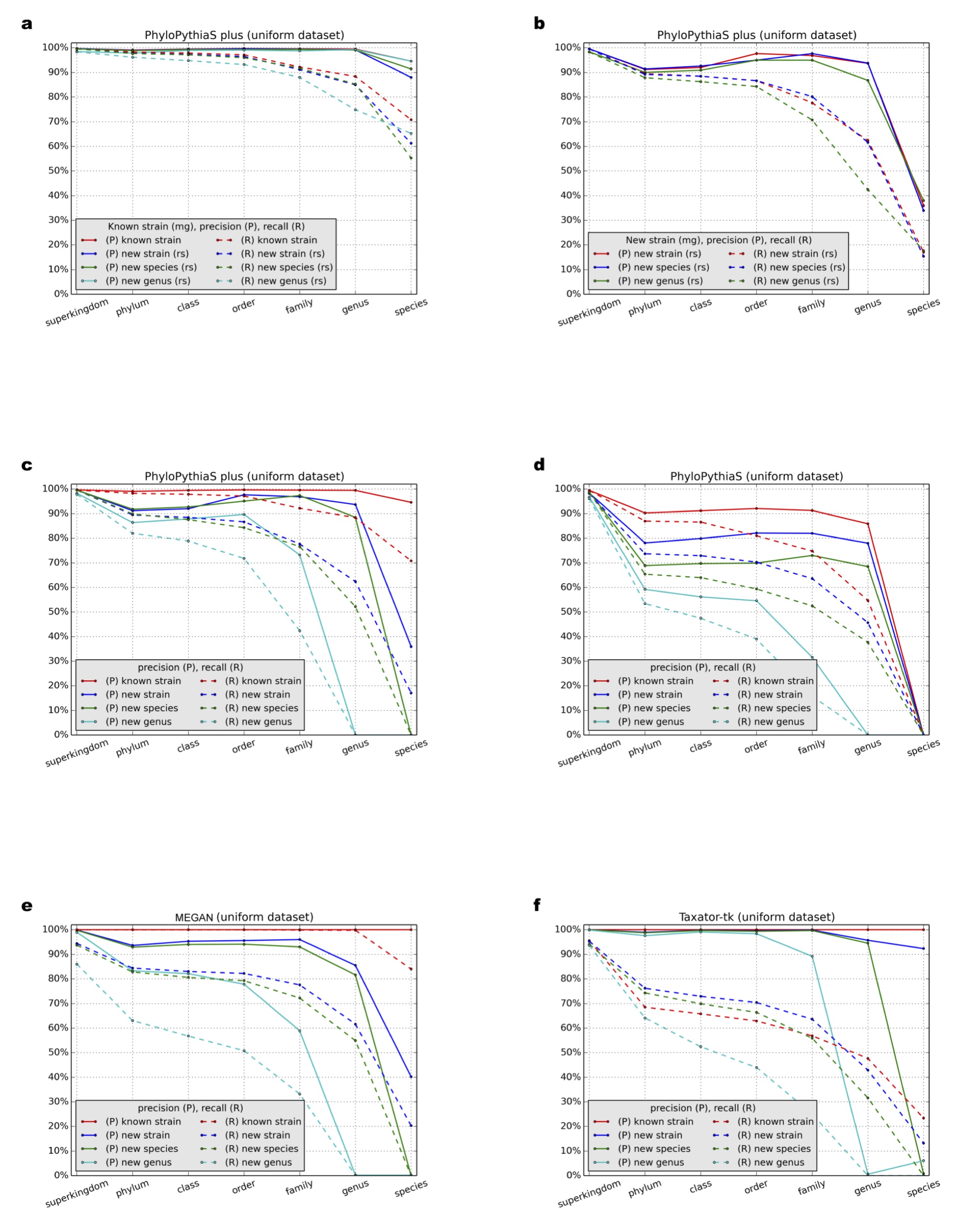

Supplement: Figure S1 — Precision (P) and recall (R) (Supplemental Information 1, Section 3.9.1) at different taxonomic ranks were calculated for (A–C) PPS+, (D) the generic PPS model, (E) MEGAN4 and (F) taxator-tk in all test scenarios (Table 1: Test Scenarios 1–9). In parentheses, (mg) and (rs) denote whether the sequences at a given taxonomic rank were masked from the marker gene or from the reference sequence collections, respectively (Supplemental Information 1, Sections 3.1 and 3.3). If not stated, sequences were masked from both reference collections. [file peerj-04-1603-s003.jpg]

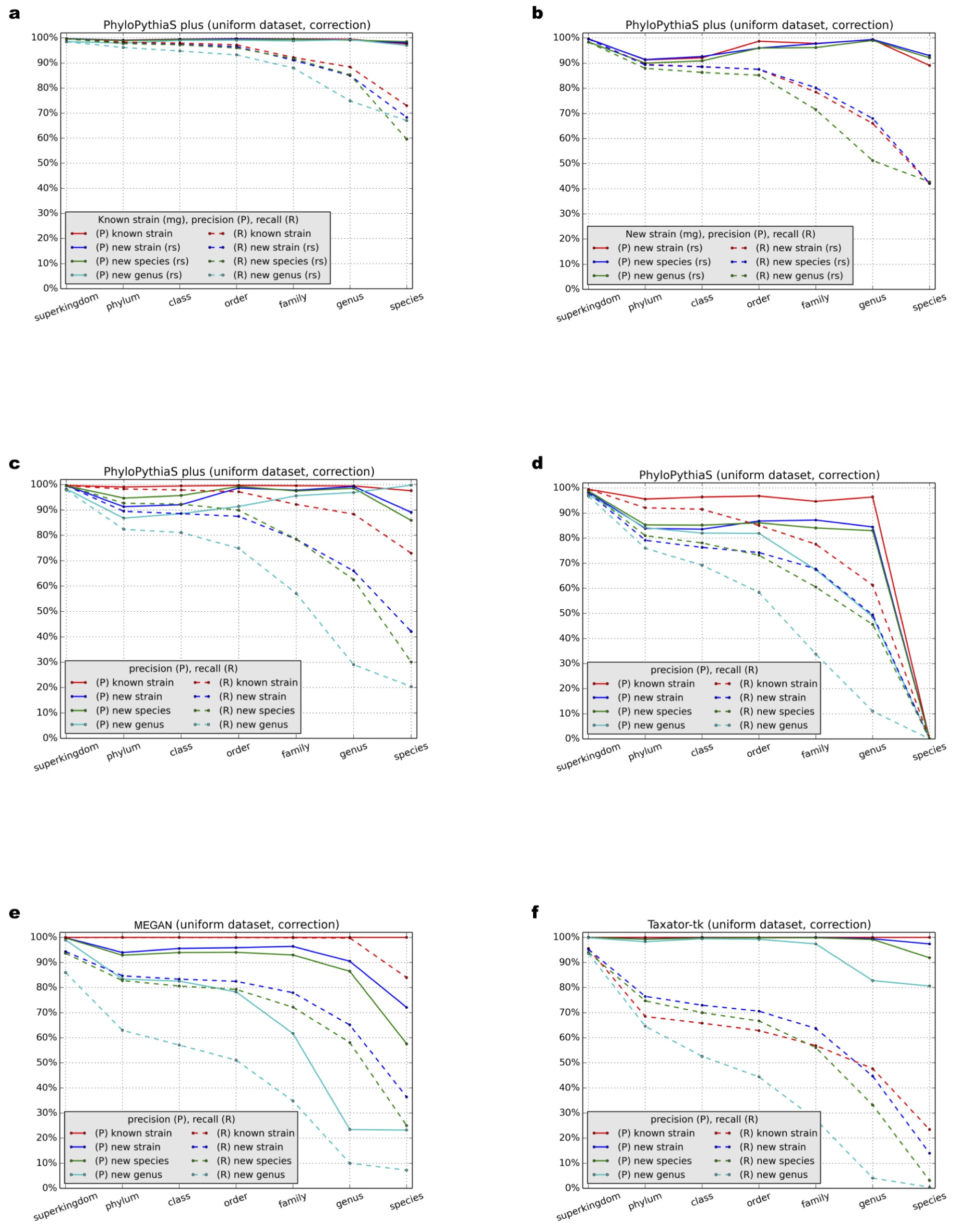

Supplement: Figure S2 — Precision (P) and recall (R) were calculated with a ‘correction’ (Supplemental Information 1, Section 3.9) at different taxonomic ranks for (A–C) PPS+, (D) the generic PPS model, (E) MEGAN4 and (F) taxator-tk in all test scenarios (Table 1: Test Scenarios 1–9, Supplemental Information 1, Section 3.1). [file peerj-04-1603-s004.jpg]

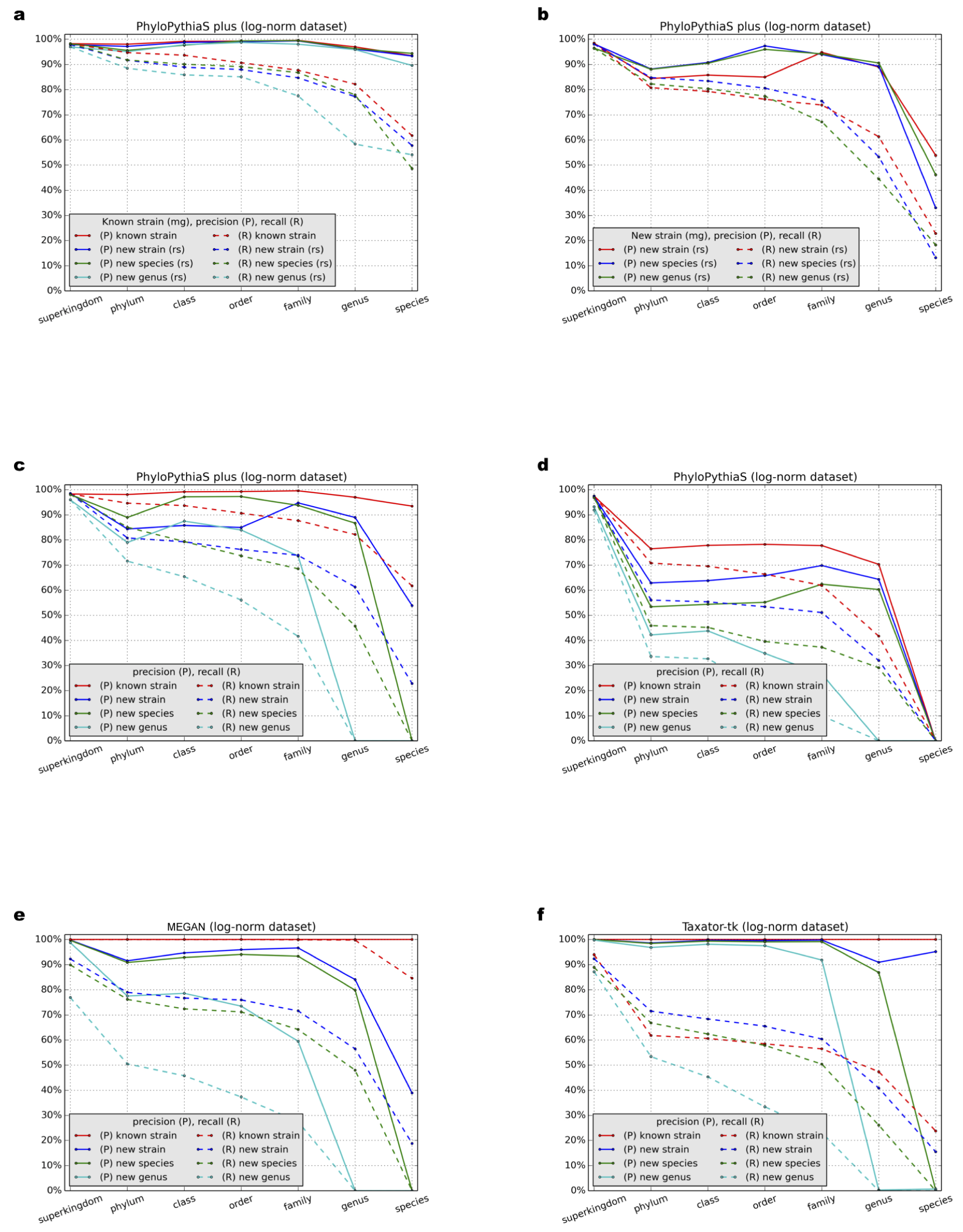

Supplement: Figure S3 — Precision (P) and recall (R) (Section 3.9.1) at different taxonomic ranks were calculated for (A–C) PPS+, (D) the generic PPS model, (E) MEGAN4 and (F) taxator-tk in all test scenarios (Table 1: Test Scenarios 1–9, Supplemental Information 1, Section 3.1). [file peerj-04-1603-s005.jpg]

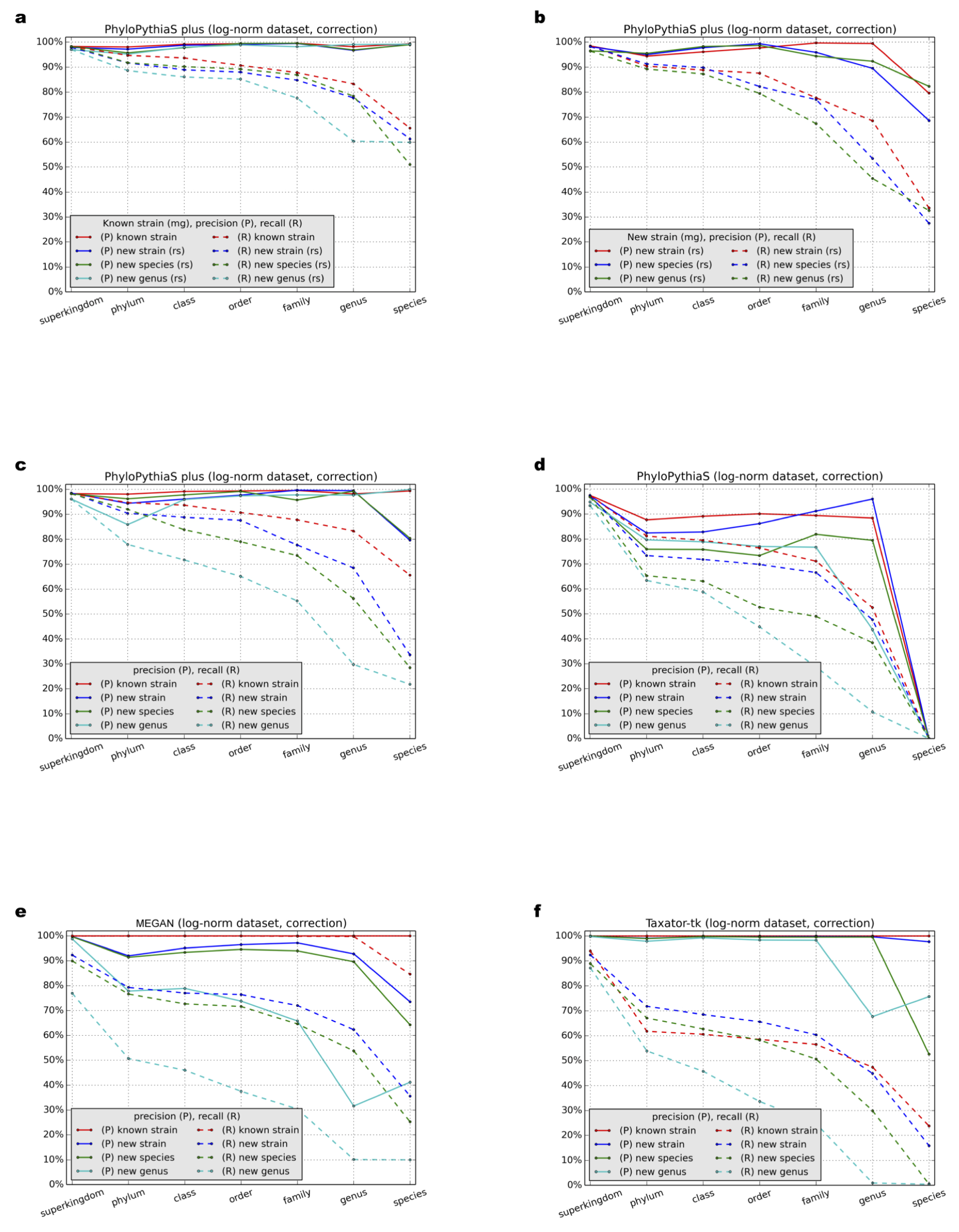

Supplement: Figure S4 — Precision (P) and recall (R) were calculated with a ‘correction’ (Supplemental Information 1, Section 3.9) at different taxonomic ranks for (A–C) PPS+, (D) the generic PPS model, (E) MEGAN4 and (F) taxator-tk in all test scenarios (Table 1: Test Scenarios 1–9, Supplemental Information 1, Section 3.1). [file peerj-04-1603-s006.jpg]

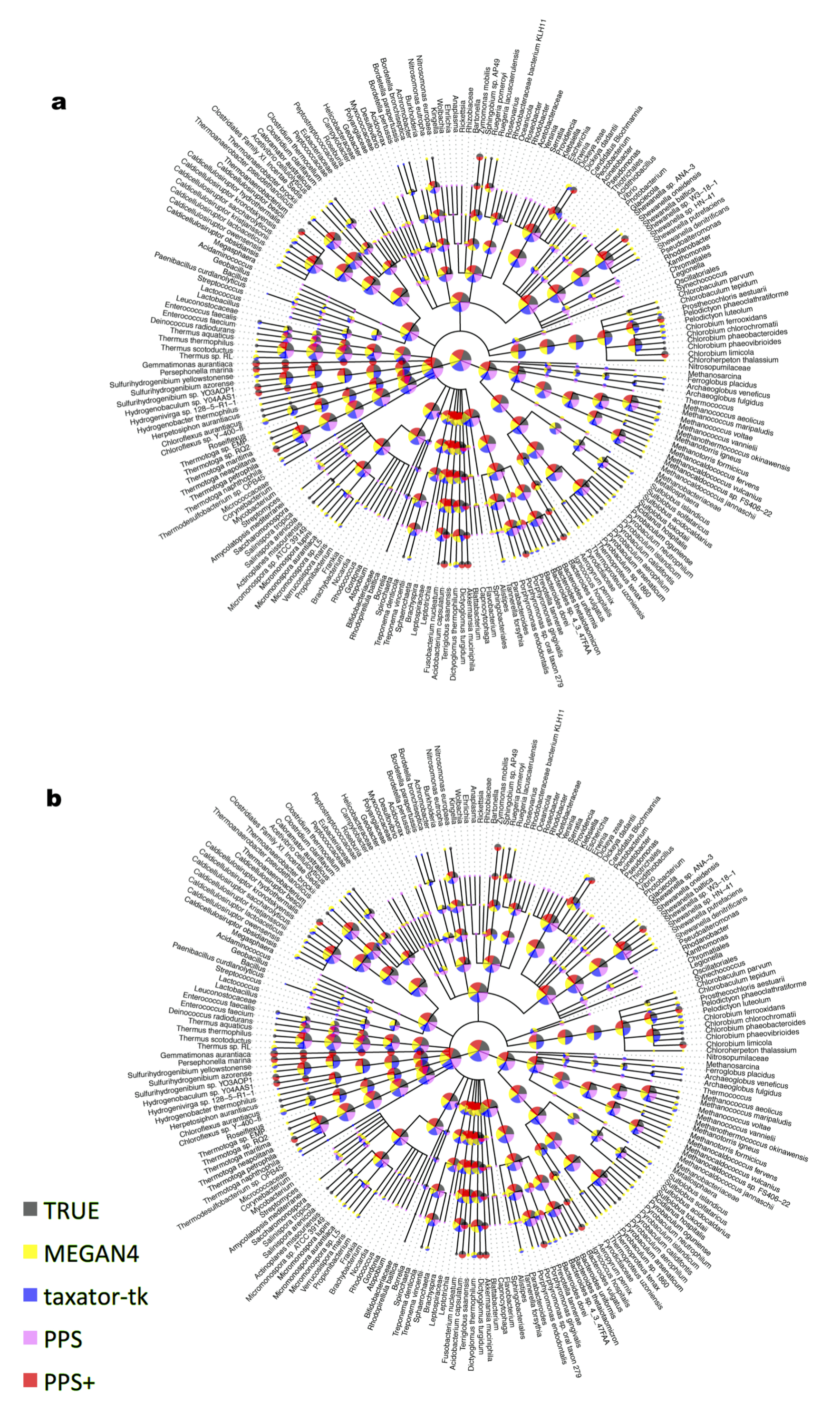

Supplement: Figure S5 — The number of taxonomic assignments to each taxon in bp is indicated on a log-scale by the pie chart sizes for PPS+, the generic PPS model, taxator-tk, MEGAN4 and the underlying standard of truth (TRUE). There were 47 strains present in the simulated metagenome sample. Assignments to taxa not shown in black in the chart are to false taxa that are not present in the simulated metagenome. (A) shows the scenario where sequences from the same species as those of the simulated dataset were excluded from the reference sequences but not the marker gene databases (Table 1: Test Scenario 3). (B) shows the scenario where sequences from the same species as those of the simulated dataset were excluded from the reference sequence and marker gene databases (Table 1: Test Scenario 8). [file peerj-04-1603-s007.jpg]

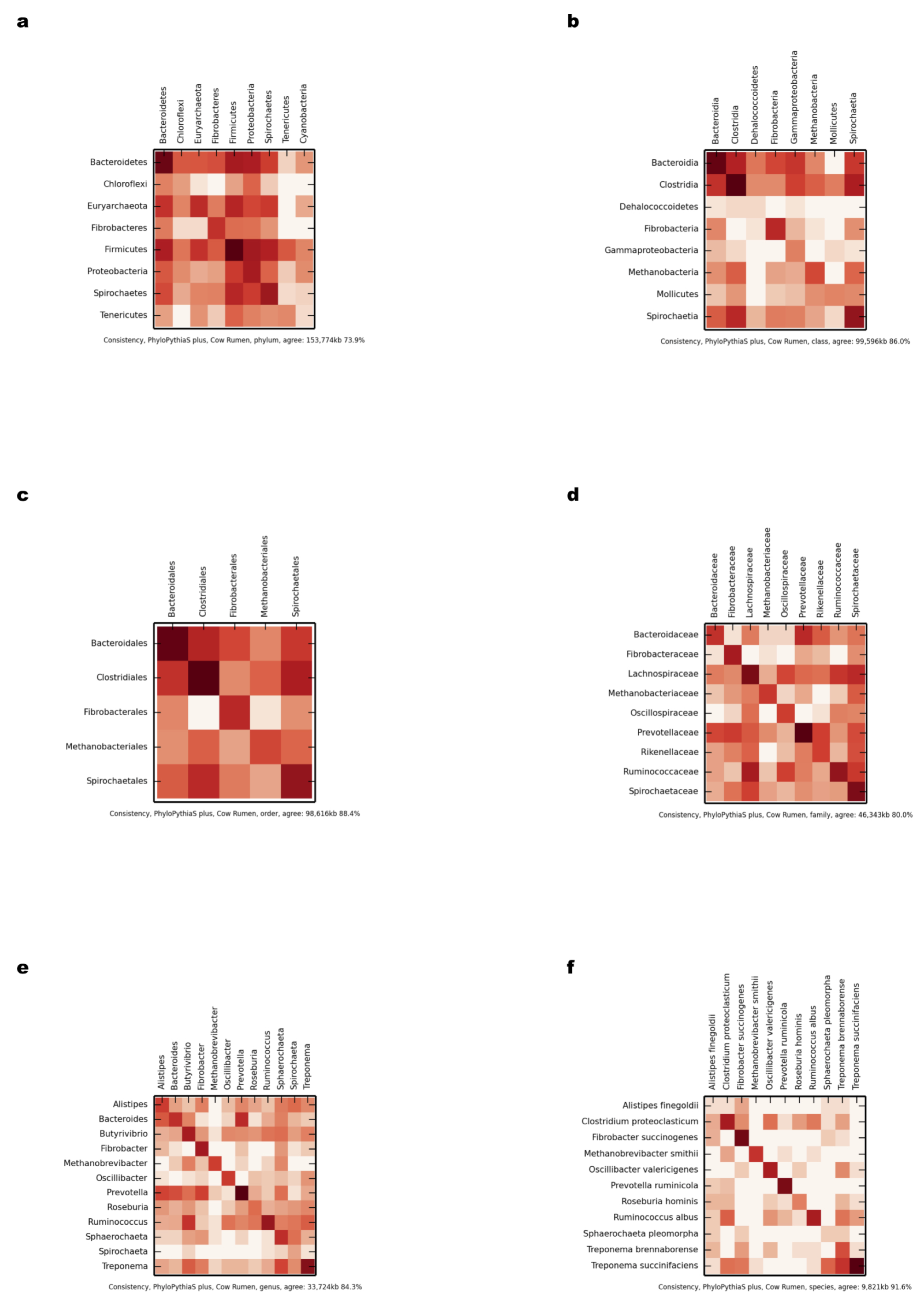

Supplement: Figure S6 — The comparisons were performed at different taxonomic ranks using heat maps (Supplemental Information 1, Sections 3.2.2 and 3.10.1). The rows correspond to scaffolds and the columns correspond to contig assignments. (A) Phylum; (B) class; (C) order; (D) family; (E) genus; (F) species. [file peerj-04-1603-s008.jpg]

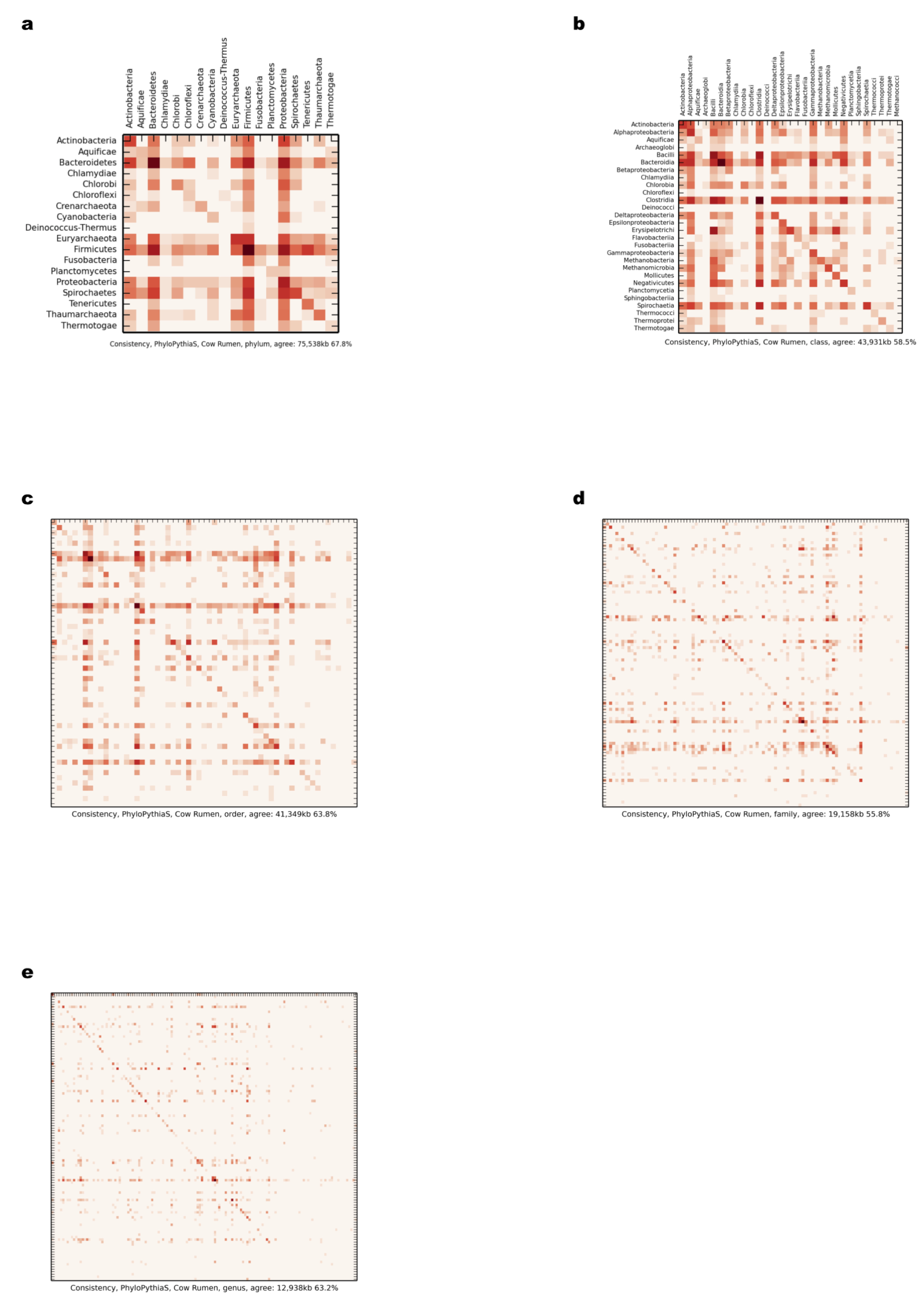

Supplement: Figure S7 — The comparisons were performed at different taxonomic ranks using heat maps (Supplemental Information 1, Sections 3.2.2 and 3.10.1). The rows correspond to scaffolds and the columns correspond to contig assignments. (A) Phylum; (B) class; (C) order; (D) family; (E) genus. [file peerj-04-1603-s009.jpg]

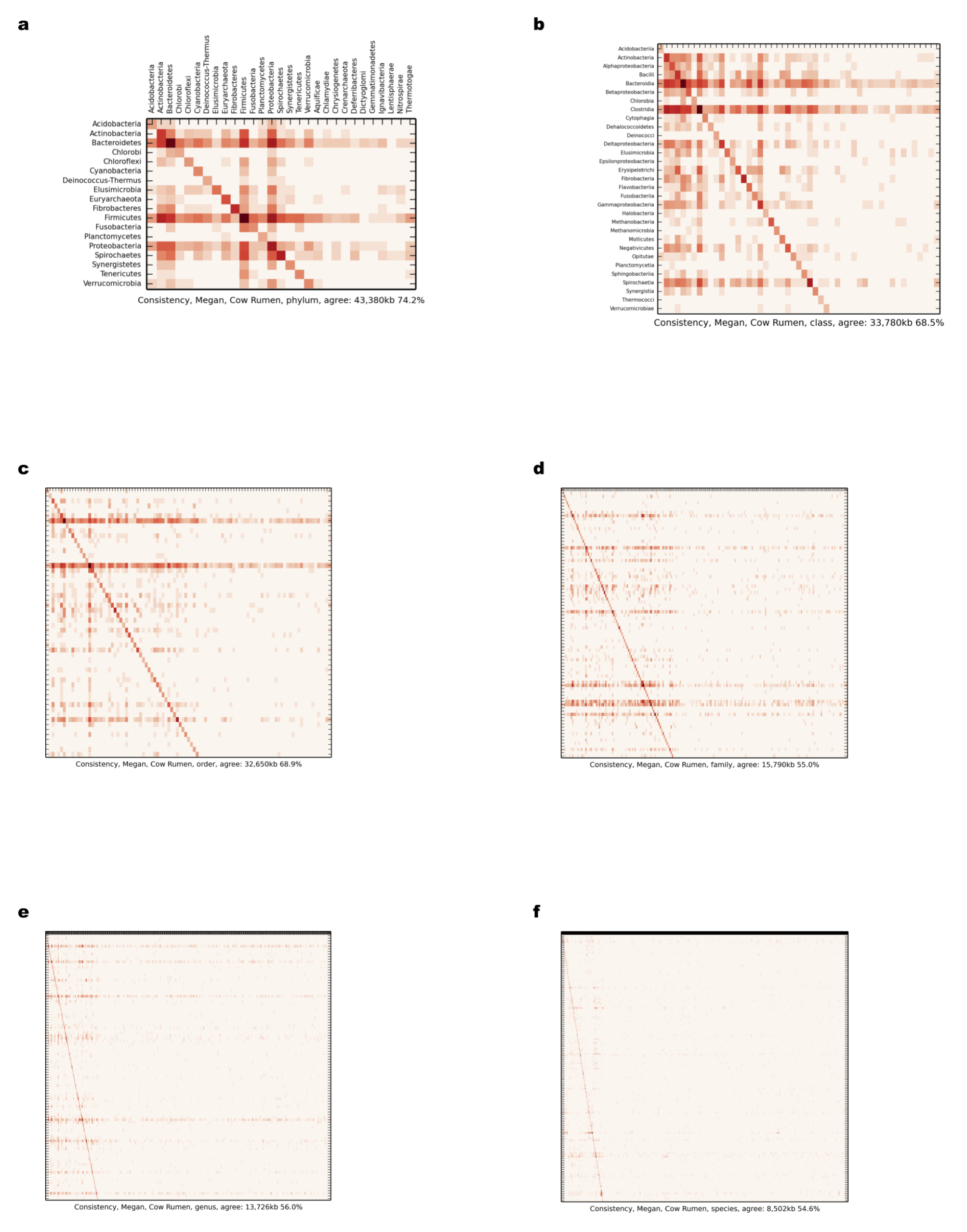

Supplement: Figure S8 — The comparisons were performed at different taxonomic ranks using heat maps (Supplemental Information 1, Section 3.2.2 and 3.10.1). The rows correspond to scaffolds and the columns correspond to contig assignments. (A) Phylum; (B) class; (C) order; (D) family; (E) genus; (F) species. [file peerj-04-1603-s010.jpg]

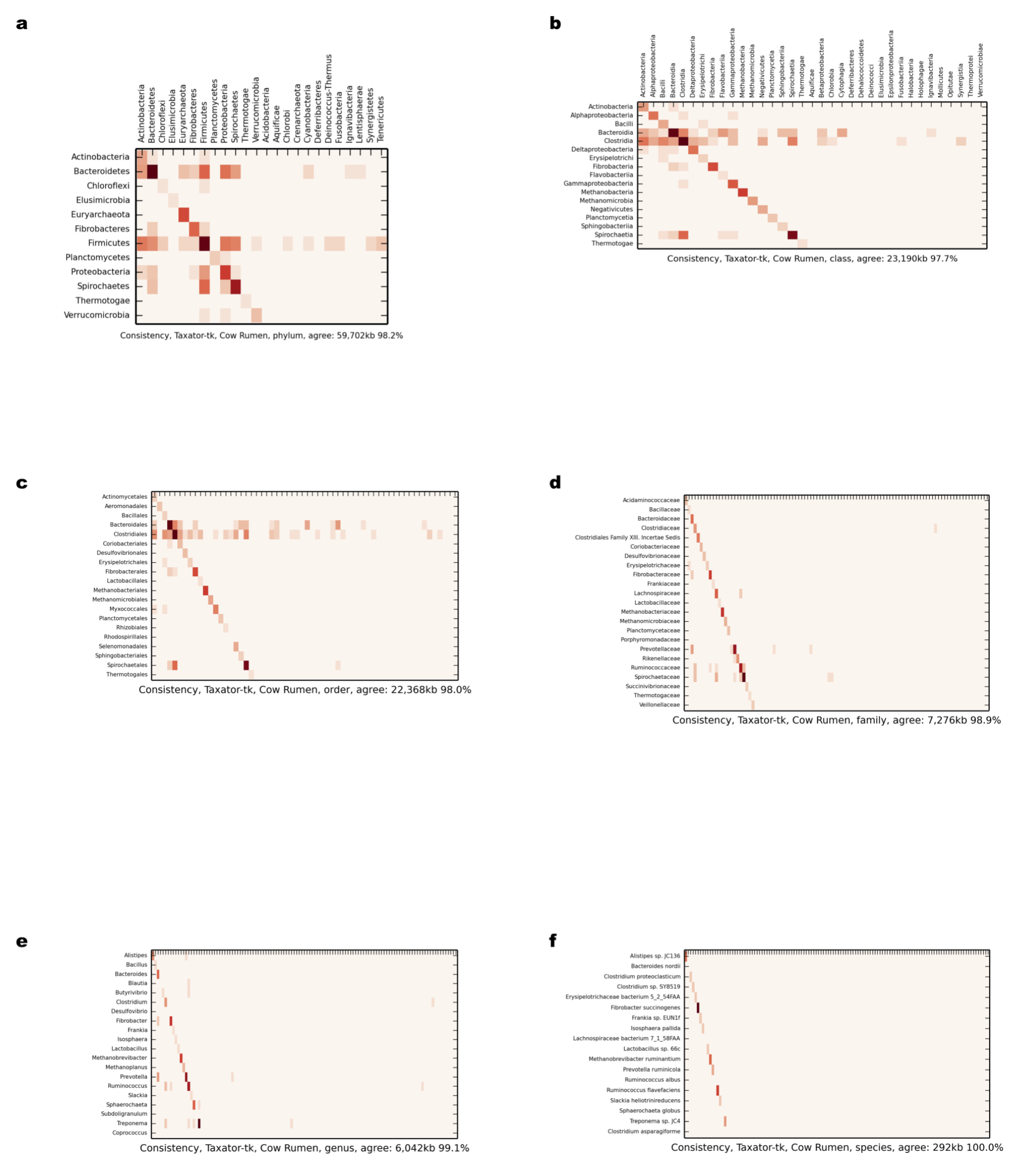

Supplement: Figure S9 — The comparisons were performed at different taxonomic ranks using heat maps (Supplemental Information 1, Section 3.2.2 and 3.10.1). The rows correspond to scaffolds and the columns correspond to contig assignments. (A) Phylum; (B) class; (C) order; (D) family; (E) genus; (F) species. [file peerj-04-1603-s011.jpg]

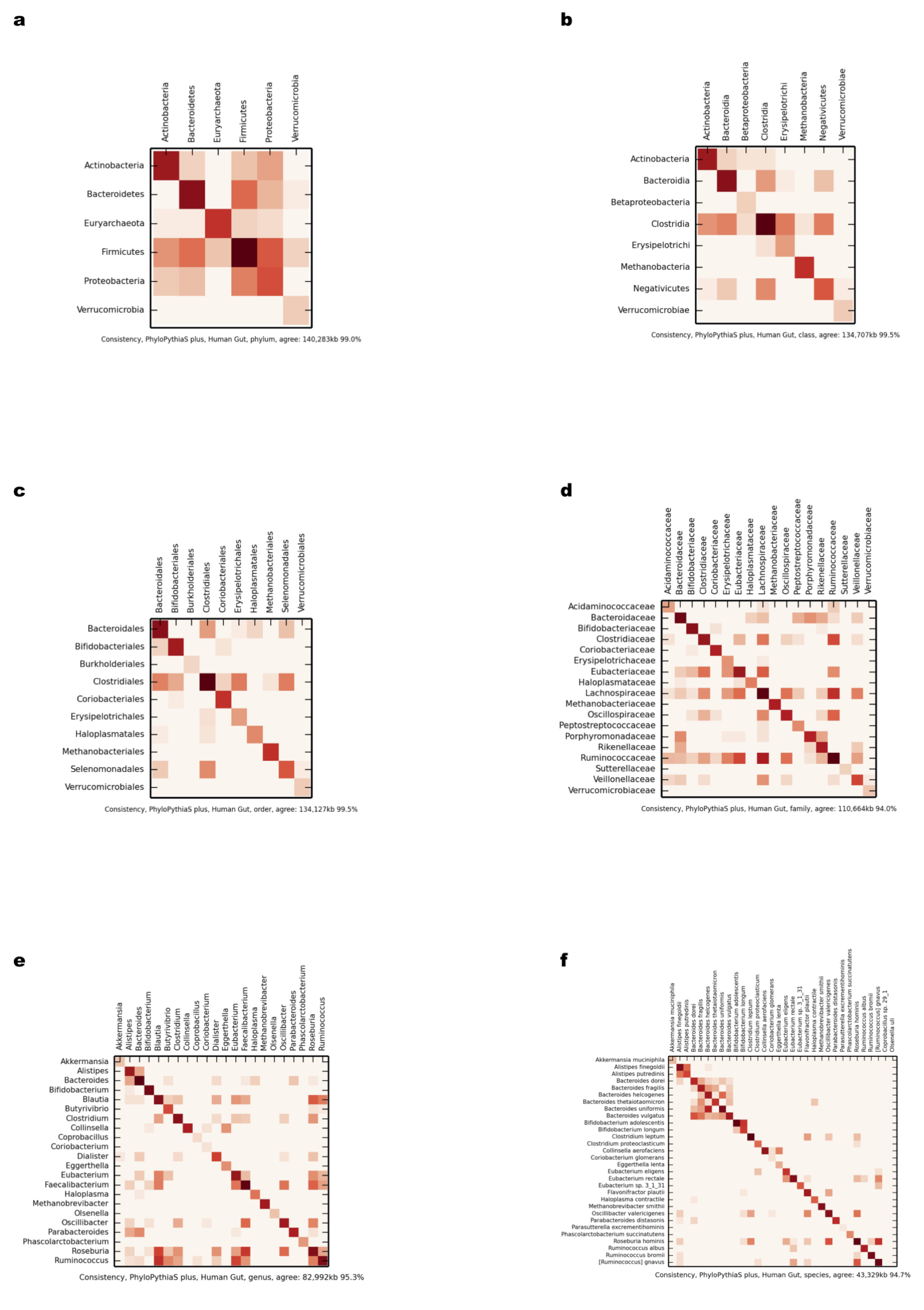

Supplement: Figure S10 — The comparisons were performed at different taxonomic ranks using heat maps (Supplemental Information 1, Sections 3.2.1 and 3.10.1). The rows correspond to scaffolds and the columns correspond to contig assignments. (A) Phylum; (B) class; (C) order; (D) family; (E) genus; (F) species. [file peerj-04-1603-s012.jpg]

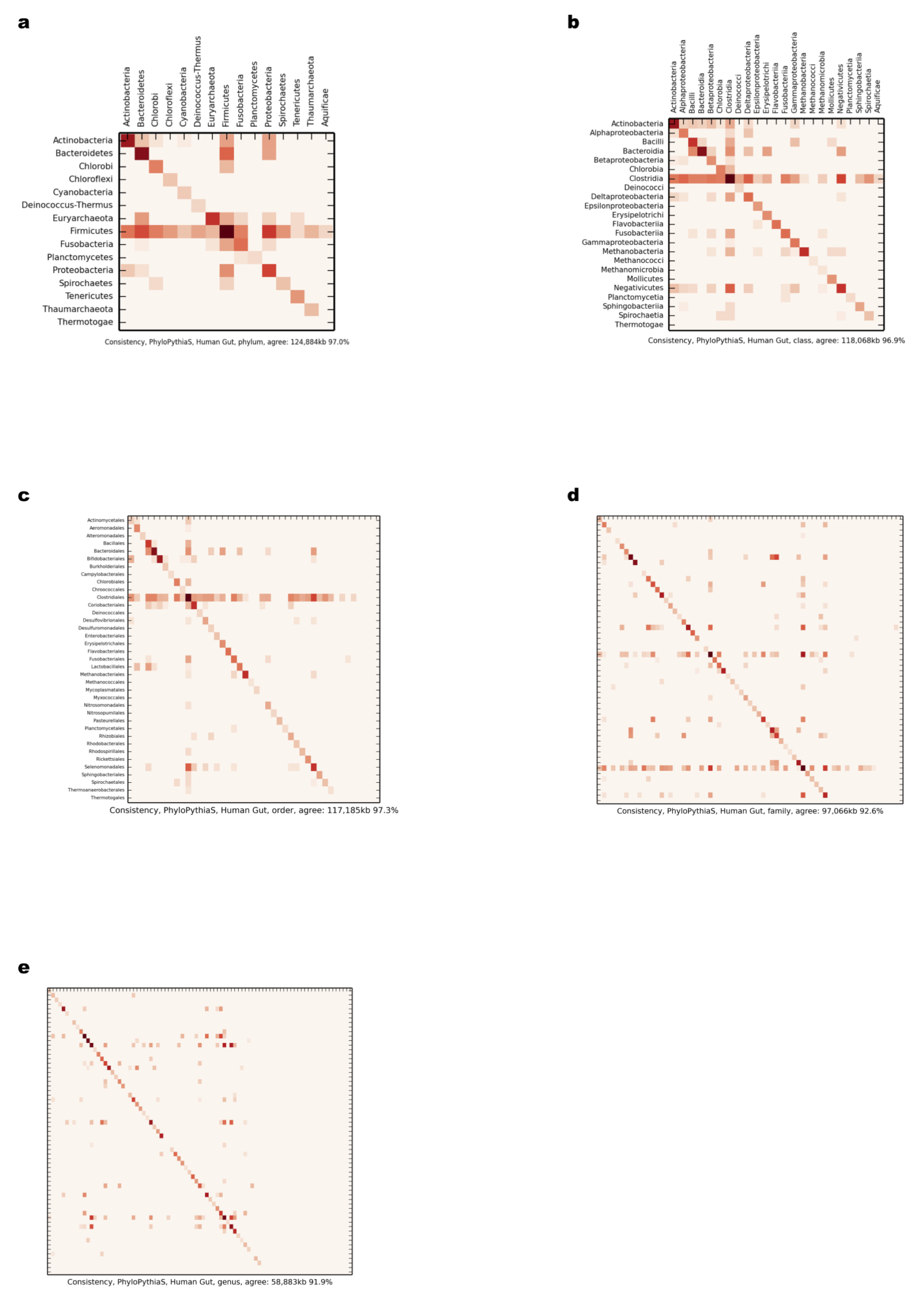

Supplement: Figure S11 — The comparisons were performed at different taxonomic ranks using heat maps (Supplemental Information 1, Sections 3.2.1 and 3.10.1). The rows correspond to scaffolds and the columns correspond to contig assignments. (A) Phylum; (B) class; (C) order; (D) family; (E) genus. [file peerj-04-1603-s013.jpg]

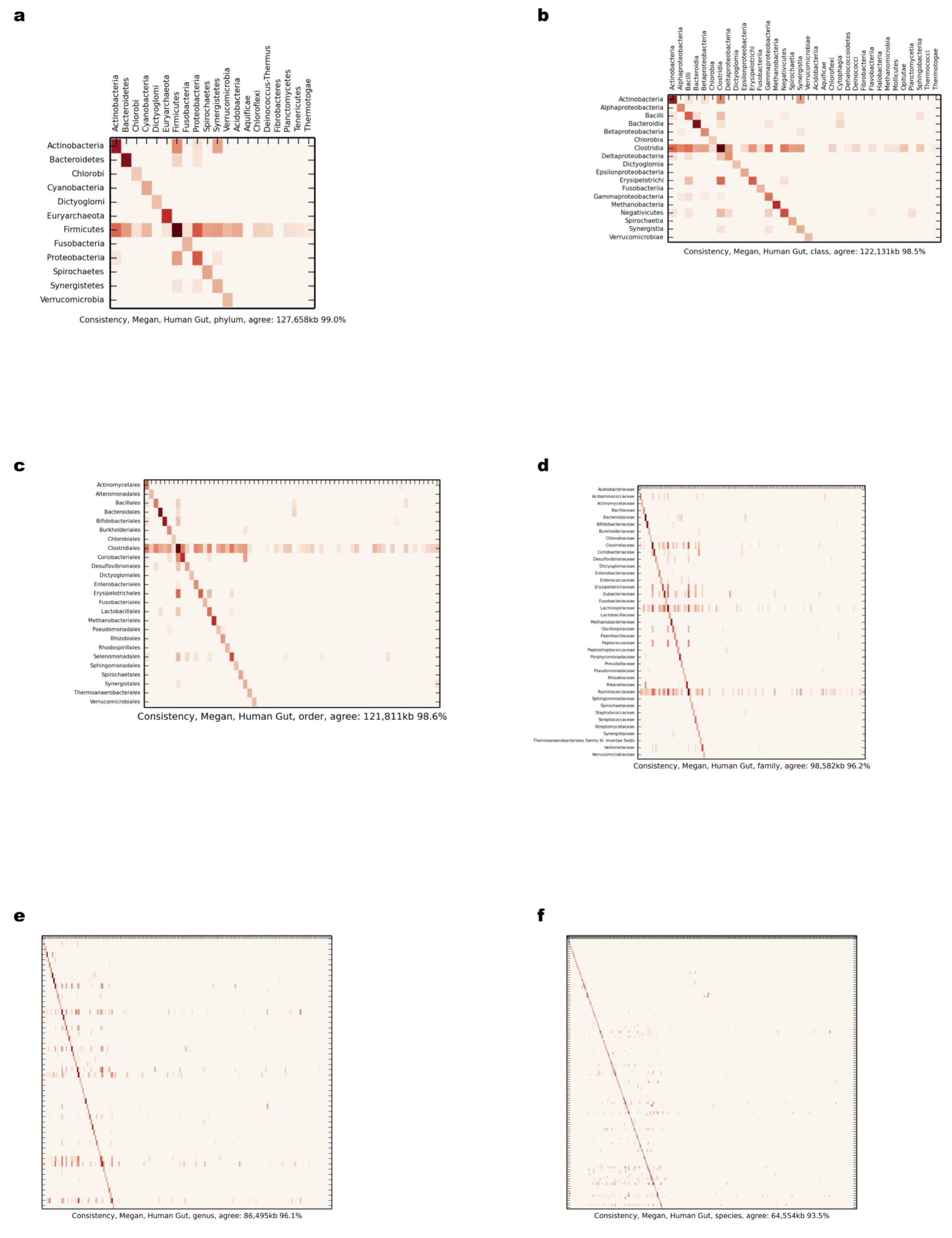

Supplement: Figure S12 — The comparisons were performed at different taxonomic ranks using heat maps (Supplemental Information 1, Sections 3.2.1 and 3.10.1). The rows correspond to scaffolds and the columns correspond to contig assignments. (A) Phylum; (B) class; (C) order; (D) family; (E) genus; (F) species. [file peerj-04-1603-s014.jpg]

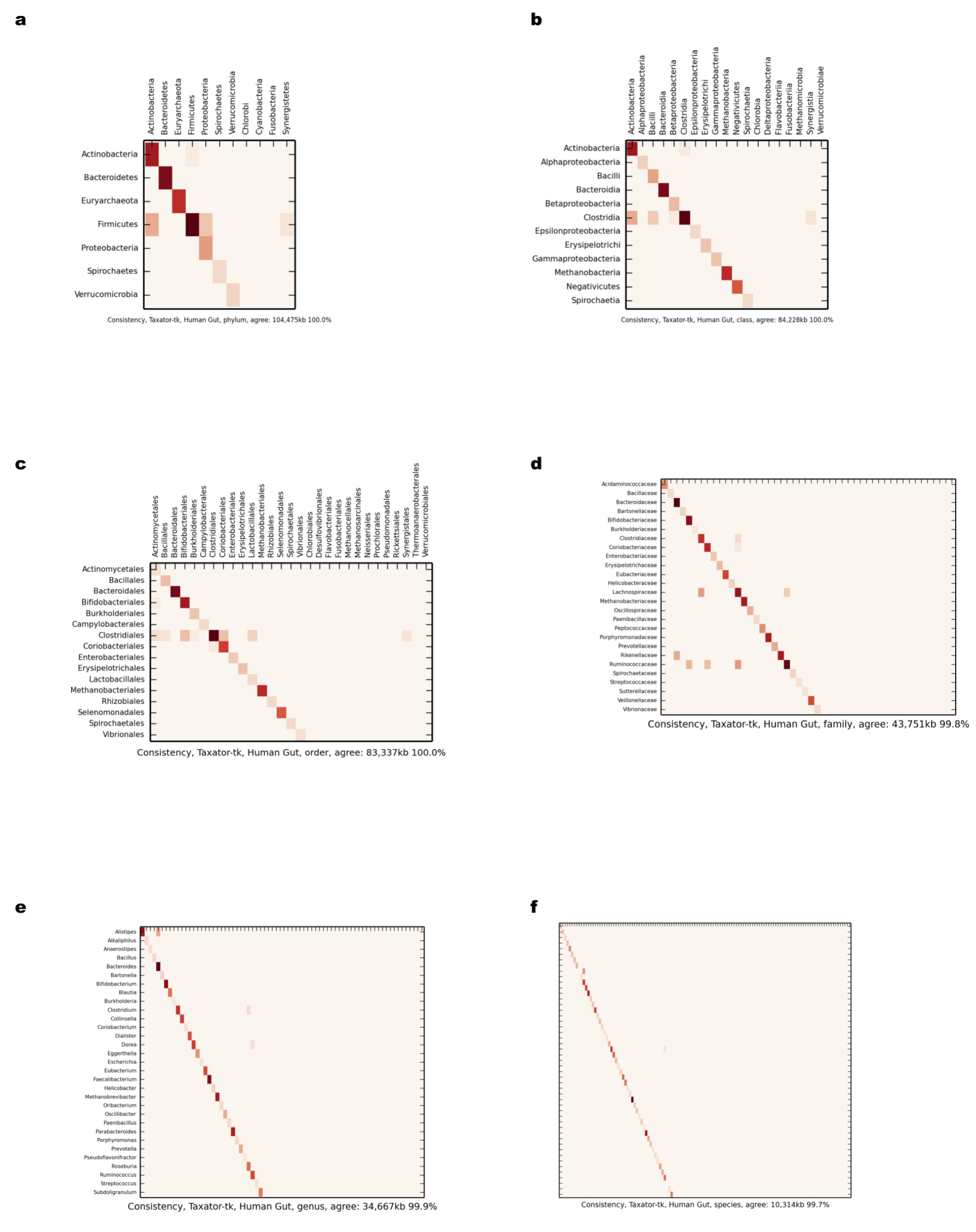

Supplement: Figure S13 — The comparisons were performed at different taxonomic ranks using heat maps (Supplemental Information 1, Sections 3.2.1 and 3.10.1). The rows correspond to scaffolds and the columns correspond to contig assignments. (A) Phylum; (B) class; (C) order; (D) family; (E) genus; (F) species. [file peerj-04-1603-s015.jpg]

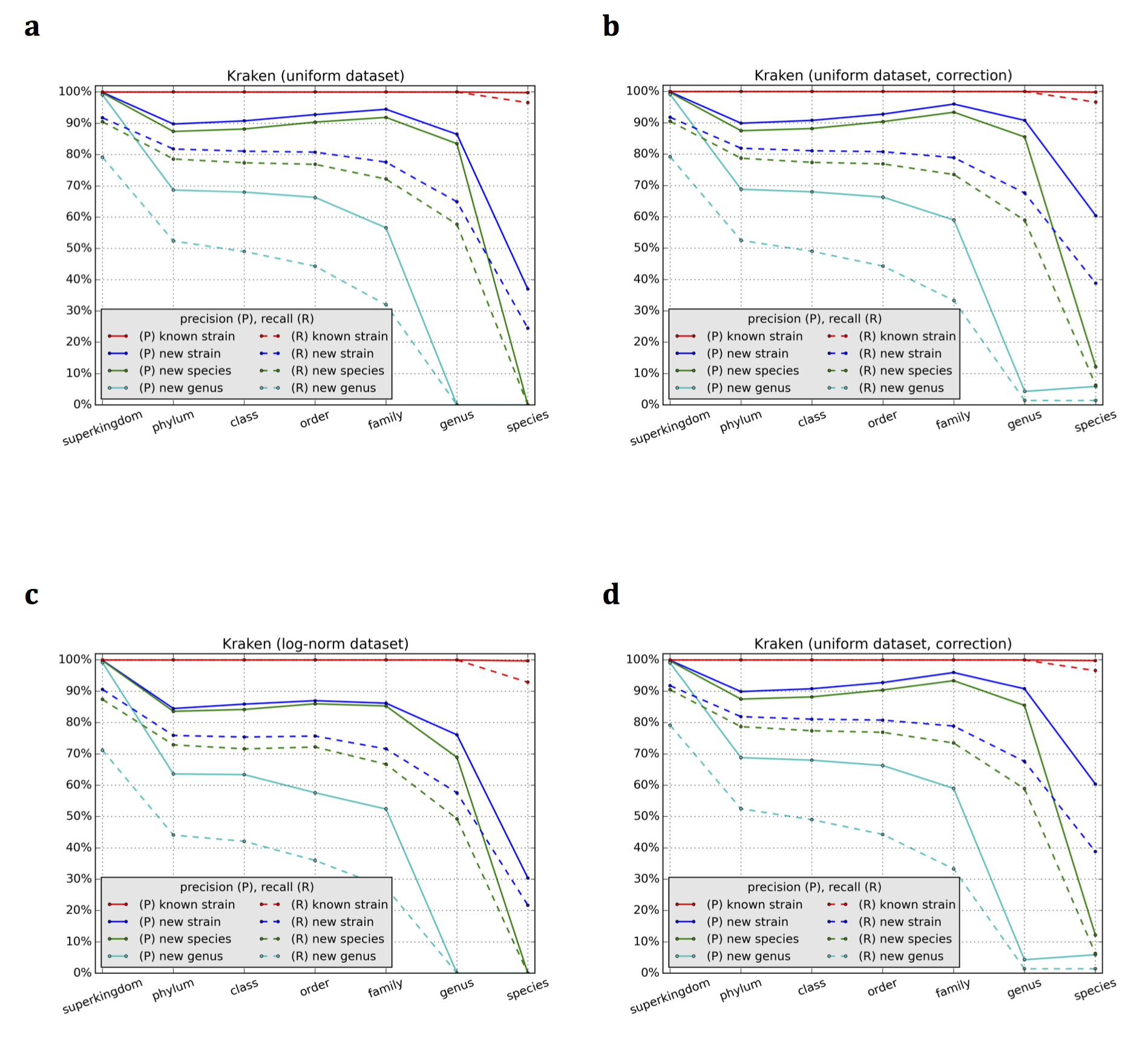

Supplement: Figure S14 — Precision (P) and recall (R) (Supplemental Information 1, Section 3.9) at different taxonomic ranks were calculated for the Kraken software in four test scenarios (Table 1: Test Scenarios 1, 5, 8, 9, Supplemental Information 1, Section 3.1) using the simulated datasets with the uniform (A and B) and log-norm (C and D) distribution. [file peerj-04-1603-s016.jpg]
