# Supplementary material for: PhyloPythiaS+: a self-training method for the rapid reconstruction of low-ranking taxonomic bins from metagenomes
Source: PeerJ. 2016 Feb 8;4:e1603. doi: 10.7717/peerj.1603 (PMC4748697; doi:10.7717/peerj.1603)
Supplement: Table S1 — The relevant combination of k-mers that is typically counted for taxonomic binning is marked in bold. The benchmark was run in one thread on a server with an Intel Xeon (CPU X5660, 2.8 GHz) processor, nevertheless we observed that Jellyfish 1.1.1 took approximately 30% more CPU resources than specified. Parallel runs of the methods can be done by splitting the input FASTA file, running multiple instances of a tool for each file separately in parallel and merging of the result files, thus the runtimes scale approximately linearly with the number of CPUs used. As a benchmark dataset, concatenated contigs from (Turnbaugh et al., 2010) (255 Mb) were used. [file peerj-04-1603-s017.docx]

| *k-*mer lengths | *PPS+* | *Jellyfish 1.1.1* | *Jellyfish 2.2* | *KAnalyze 0.9.7* |
| --- | --- | --- | --- | --- |
| 4 | 7.5 s | 6.2 s | 21.7 s | 29.5 s |
| 5 | 7.5 s | 6.2 s | 22.0 s | 34.7 s |
| 6 | 7.5 s | 6.2 s | 21.9 s | 39.1 s |
| **4, 5, 6** | **9.0 s** | **18.6 s** | **1m 5 s** | **1m 43 s** |
| 7 | 7.6 s | 6.2 s | 22.4 s | 43.9 s |
| 8 | 8.0 s | 6.2 s | 23.0 s | 48.9 s |
| 9 | 8.4 s | 6.3 s | 24.6 s | 54.4 s |
| 7, 8, 9 | 11.1 s | 18.7 s | 1 m 10 s | 2 m 27 s |
