# Supplementary material for: PhyloPythiaS+: a self-training method for the rapid reconstruction of low-ranking taxonomic bins from metagenomes
Source: PeerJ. 2016 Feb 8;4:e1603. doi: 10.7717/peerj.1603 (PMC4748697; doi:10.7717/peerj.1603)
Supplement: Table S2 [file peerj-04-1603-s018.docx]

| Method | Rank | F_1_-score (%) | Precision (%) | Recall = Correct (%) | Incorrect (%) | Unassigned (%) |
| --- | --- | --- | --- | --- | --- | --- |
| *taxator-tk* | Family | 66.6 | 98.2 | 50.4 | 0.9 | 48.7 |
| *PPS* | Family | 60.4 | 72.6 | 51.7 | 19.5 | 28.8 |
| *MEGAN* | Family | 78.8 | 88.9 | 70.7 | 8.8 | 20.4 |
| *Kraken* | Family | 74.7 | 79.6 | 70.4 | 18.0 | 11.5 |
| *PPS+* | Family | 88.4 | 96.4 | 81.6 | 3.0 | 15.4 |
| *taxator-tk* | Genus | 46.1 | 93.2 | 30.6 | 2.2 | 67.2 |
| *PPS* | Genus | 45.8 | 68.2 | 34.5 | 16.1 | 49.4 |
| *MEGAN* | Genus | 63.1 | 75.7 | 54.1 | 17.4 | 28.5 |
| *Kraken* | Genus | 59.3 | 63.4 | 55.7 | 32.1 | 12.2 |
| *PPS+* | Genus | 77.4 | 91.8 | 66.9 | 6.0 | 27.1 |
| *taxator-tk* | Species | 16.7 | 87.8 | 9.2 | 1.3 | 89.6 |
| *PPS* | Species | N/A | N/A | N/A | N/A | 100.0 |
| *MEGAN* | Species | 34.2 | 49.6 | 26.1 | 26.5 | 47.4 |
| *Kraken* | Species | 32.8 | 35.7 | 30.3 | 54.6 | 15.2 |
| *PPS+* | Species | 51.5 | 71.4 | 40.3 | 16.1 | 43.6 |
