# Supplementary material for: PhyloPythiaS+: a self-training method for the rapid reconstruction of low-ranking taxonomic bins from metagenomes
Source: PeerJ. 2016 Feb 8;4:e1603. doi: 10.7717/peerj.1603 (PMC4748697; doi:10.7717/peerj.1603)
Supplement: Table S3 [file peerj-04-1603-s019.docx]

| Method | Rank | F_1_-score (%) | Precision (%) | Recall = Correct (%) | Incorrect (%) | Unassigned (%) |
| --- | --- | --- | --- | --- | --- | --- |
| *taxator-tk* | Family | 67.4 | 99.4 | 51.0 | 0.3 | 48.7 |
| *PPS* | Family | 70.0 | 84.2 | 59.9 | 11.3 | 28.8 |
| *MEGAN* | Family | 79.3 | 89.5 | 71.2 | 8.3 | 20.4 |
| *Kraken* | Family | 75.8 | 80.7 | 71.4 | 17.1 | 11.5 |
| *PPS+* | Family | 90.1 | 98.3 | 83.2 | 1.5 | 15.4 |
| *taxator-tk* | Genus | 48.8 | 98.9 | 32.4 | 0.4 | 67.2 |
| *PPS* | Genus | 55.5 | 82.7 | 41.8 | 8.8 | 49.4 |
| *MEGAN* | Genus | 68.0 | 81.5 | 58.3 | 13.2 | 28.5 |
| *Kraken* | Genus | 60.7 | 64.9 | 57.0 | 30.8 | 12.2 |
| *PPS+* | Genus | 83.2 | 98.6 | 71.9 | 1.0 | 27.1 |
| *taxator-tk* | Species | 18.5 | 98.2 | 10.2 | 0.2 | 89.6 |
| *PPS* | Species | N/A | N/A | N/A | N/A | 100.0 |
| *MEGAN* | Species | 50.0 | 72.5 | 38.1 | 14.4 | 47.4 |
| *Kraken* | Species | 38.7 | 42.2 | 35.8 | 49.1 | 15.2 |
| *PPS+* | Species | 68.9 | 95.5 | 53.9 | 2.5 | 43.6 |
