# Supplementary material for: PhyloPythiaS+: a self-training method for the rapid reconstruction of low-ranking taxonomic bins from metagenomes
Source: PeerJ. 2016 Feb 8;4:e1603. doi: 10.7717/peerj.1603 (PMC4748697; doi:10.7717/peerj.1603)
Supplement: Table S4 [file peerj-04-1603-s020.docx]

| Method | Rank | F_1_-score (%) | Precision (%) | Recall = Correct (%) | Incorrect (%) | Unassigned (%) |
| --- | --- | --- | --- | --- | --- | --- |
| *taxator-tk* | Family | 64.2 | 98.5 | 47.6 | 0.7 | 51.7 |
| *PPS* | Family | 49.2 | 63.5 | 40.1 | 23.0 | 36.9 |
| *MEGAN* | Family | 76.3 | 90.7 | 65.8 | 6.8 | 27.4 |
| *Kraken* | Family | 71.8 | 78.1 | 66.4 | 18.6 | 15.0 |
| *PPS+* | Family | 85.0 | 95.7 | 76.5 | 3.4 | 20.0 |
| *taxator-tk* | Genus | 43.7 | 92.3 | 28.6 | 2.4 | 69.0 |
| *PPS* | Genus | 35.2 | 56.0 | 25.7 | 20.2 | 54.1 |
| *MEGAN* | Genus | 61.9 | 78.6 | 51.1 | 13.9 | 35.0 |
| *Kraken* | Genus | 56.0 | 61.1 | 51.7 | 33.0 | 15.3 |
| *PPS+* | Genus | 72.9 | 90.1 | 61.2 | 6.7 | 32.1 |
| *taxator-tk* | Species | 17.8 | 94.1 | 9.8 | 0.6 | 89.6 |
| *PPS* | Species | N/A | N/A | N/A | N/A | 100.0 |
| *MEGAN* | Species | 34.6 | 52.3 | 25.9 | 23.6 | 50.5 |
| *Kraken* | Species | 31.6 | 35.4 | 28.6 | 52.4 | 19.0 |
| *PPS+* | Species | 48.9 | 73.1 | 36.7 | 13.5 | 49.8 |
