# Supplementary material for: PhyloPythiaS+: a self-training method for the rapid reconstruction of low-ranking taxonomic bins from metagenomes
Source: PeerJ. 2016 Feb 8;4:e1603. doi: 10.7717/peerj.1603 (PMC4748697; doi:10.7717/peerj.1603)
Supplement: Table S5 [file peerj-04-1603-s021.docx]

| Method | Rank | F_1_-score (%) | Precision (%) | Recall = Correct (%) | Incorrect (%) | Unassigned (%) |
| --- | --- | --- | --- | --- | --- | --- |
| *taxator-tk* | Family | 64.8 | 99.4 | 48.1 | 0.3 | 51.7 |
| *PPS* | Family | 66.1 | 85.4 | 53.9 | 9.2 | 36.9 |
| *MEGAN* | Family | 77.3 | 92.0 | 66.7 | 5.8 | 27.4 |
| *Kraken* | Family | 72.5 | 78.9 | 67.1 | 17.9 | 15.0 |
| *PPS+* | Family | 87.5 | 98.5 | 78.7 | 1.2 | 20.0 |
| *taxator-tk* | Genus | 47.0 | 99.3 | 30.8 | 0.2 | 69.0 |
| *PPS* | Genus | 51.2 | 81.4 | 37.3 | 8.5 | 54.1 |
| *MEGAN* | Genus | 68.5 | 86.9 | 56.5 | 8.5 | 35.0 |
| *Kraken* | Genus | 56.8 | 61.9 | 52.4 | 32.3 | 15.3 |
| *PPS+* | Genus | 78.7 | 97.3 | 66.1 | 1.9 | 32.1 |
| *taxator-tk* | Species | 18.5 | 97.3 | 10.2 | 0.3 | 89.6 |
| *PPS* | Species | N/A | N/A | N/A | N/A | 100.0 |
| *MEGAN* | Species | 52.0 | 78.6 | 38.8 | 10.6 | 50.5 |
| *Kraken* | Species | 39.3 | 43.9 | 35.6 | 45.5 | 19.0 |
| *PPS+* | Species | 62.2 | 93.1 | 46.7 | 3.5 | 49.8 |
