# Supplementary material for: PhyloPythiaS+: a self-training method for the rapid reconstruction of low-ranking taxonomic bins from metagenomes
Source: PeerJ. 2016 Feb 8;4:e1603. doi: 10.7717/peerj.1603 (PMC4748697; doi:10.7717/peerj.1603)
Supplement: Table S6 — Contigs of the cow rumen dataset of at least 10 kb were divided into chunks of 2 kb for evaluation of assignment consistency (Supplemental Information 1, Section 3.2.2). Scaffold-contig consistency of the assignments made by PPS+, the generic PPS model, MEGAN4, Kraken and taxator-tk for the chunked cow rumen dataset, computed via different definitions (Supplemental Information 1, Section 3.10.2). The table also contains the number of kb of contigs assigned at low taxonomic ranks (family, genus and species) and the corresponding consistency (% agreement) (Supplemental Information 1, Section 3.10.1). Bold numbers correspond to the best values, whereas italic numbers indicate the worst values. [file peerj-04-1603-s022.docx]

| Measure | *PPS+* | *PPS* | *MEGAN4* | *Kraken* | *taxator-tk* | Def. |
| --- | --- | --- | --- | --- | --- | --- |
| Scaffolds considered | 12,192 | 12,192 | 9456 | 7859 | 11,447 |  |
| Consistent contigs  /  total contigs | 128,685  /  159,263 | 137,747  /  159,263 | 116,726  /  135,362 | 104,633  /  119,939 | 151,585  /  153,185 | 1 |
| Consistent count % | *80.80* | 86.49 | 86.23 | 87.24 | **98.96** | 1 |
| Consistent kbp  /  total kbp | 257,370  /  318,526 | 275,494  /  318,526 | 233,452  /  270,724 | 209,266  /  239,878 | 303,170  /  306,370 | 2 |
| Consistent bp % | *80.80* | 86.49 | 86.23 | 87.24 | **98.96** | 2 |
| Avg. distance to path | 0.38 | 0.30 | 0.50 | *0.60* | **0.02** | 3 |
| Avg. weighted distance to path | 0.38 | 0.30 | 0.50 | *0.60* | **0.02** | 4 |
| Avg. distance to scaffold label | 3.16 | 3.43 | 5.89 | *7.23* | **2.65** | 5 |
| Avg. weighted distance to scaffold label | 3.16 | 3.43 | 5.89 | *7.23* | **2.65** | 6 |
| Family: contigs (kb assigned) | **71,660** | 43,118 | 55,904 | 45,752 | *13,626* |  |
| Family: consistency ‘% agreement’ | 80.0 | 55.8 | 55.0 | *45.2* | **98.9** | 0b |
| Genus: contigs (kb assigned) | **53,705** | 28,077 | 53,008 | 44,600 | *10,596* |  |
| Genus: consistency ‘% agreement’ | 84.3 | 63.2 | 56.0 | *43.7* | **99.1** | 0b |
| Species: contigs (kb assigned) | 26,121 | N/A | 41,204 | **42,626** | *1426* |  |
| Species: consistency ‘% agreement’ | 91.6 | N/A | 54.6 | *38.1* | **100.0** | 0b |
