# Supplementary material for: PhyloPythiaS+: a self-training method for the rapid reconstruction of low-ranking taxonomic bins from metagenomes
Source: PeerJ. 2016 Feb 8;4:e1603. doi: 10.7717/peerj.1603 (PMC4748697; doi:10.7717/peerj.1603)
Supplement: Table S7 — Scaffold-contig consistency of the assignments made by PPS+, the generic PPS model, MEGAN4, Kraken and taxator-tk of the human gut dataset (Supplemental Information 1, Section 3.2.1) computed using different definitions (Supplemental Information 1, Section 3.10). Bold numbers correspond to the best values, whereas italic numbers indicate the worst values. [file peerj-04-1603-s023.docx]

| Measure | *PPS+* | *PPS* | *MEGAN4* | *Kraken* | *taxator-tk* | Def. |
| --- | --- | --- | --- | --- | --- | --- |
| Scaffolds considered | 47,983 | 47,983 | 83,973 | 75,926 | 99,202 |  |
| Consistent contigs  /  total contigs | 64,197  /  66,480 | 63,954  /  66,480 | 99,647  /  101,613 | 88,214  /  92,900 | 117,576  /  117,630 | 1 |
| Consistent count % | 96.57 | *96.20* | 98.07 | 94.96 | **99.95** | 1 |
| Consistent kbp  /  total kbp | 181,207  /  189,517 | 179,798  /  189,517 | 191,429  /  200,478 | 166,075  /  190,001 | 217,517  /  217,720 | 2 |
| Consistent bp % | 95.62 | 94.87 | 95.49 | *87.41* | **99.91** | 2 |
| Avg. distance to path | 0.06 | 0.07 | 0.05 | *0.14* | **0** | 3 |
| Avg. weighted distance to path | 0.07 | 0.10 | 0.12 | *0.35* | **0** | 4 |
| Avg. distance to scaffold label | 0.63 | *0.72* | 0.38 | 0.61 | **0.29** | 5 |
| Avg. weighted distance to scaffold label | **0.53** | 0.58 | 0.73 | *1.15* | 0.62 | 6 |
| Family: contigs (kb assigned) | 146,046 | 118,679 | 161,452 | **173,238** | *74,793* |  |
| Family: consistency ‘% agreement’ | 94.0 | 92.6 | 96.2 | *53.4* | **99.8** | 0b |
| Genus: contigs (kb assigned) | 110,762 | 71,934 | 149,448 | **159,556** | *61,242* |  |
| Genus: consistency ‘% agreement’ | 95.3 | 91.9 | 96.1 | *88.3* | **99.9** | 0b |
| Species: contigs (kb assigned) | 61,969 | N/A | 114,716 | **162,726** | *20,687* |  |
| Species: consistency ‘% agreement’ | 94.7 | N/A | *93.5* | 81.3 | **99.7** | 0b |
