# Supplementary material for: PhyloPythiaS+: a self-training method for the rapid reconstruction of low-ranking taxonomic bins from metagenomes
Source: PeerJ. 2016 Feb 8;4:e1603. doi: 10.7717/peerj.1603 (PMC4748697; doi:10.7717/peerj.1603)
Supplement: Dataset S1 [file peerj-04-1603-s024.pdf]

Supplemental Datasets S1–S6

PhyloPythiaS+: A Self-Training Method for the Rapid Reconstruction of Low-Ranking Taxonomic Bins from Metagenomes

This document describes how to configure the software to reproduce the results.

Download datasets

The datasets for the benchmarks can be downloaded from:
https://github.com/algbioi/datasets

Supplemental Dataset S1: Simulated dataset with uniform distribution.

Supplemental Dataset S2: Simulated dataset with log-normal distribution.

Supplemental Dataset S3: Contigs of the real chunked cow rumen dataset.

Supplemental Dataset S4: Scaffolds of the real chunked cow rumen dataset.

Supplemental Dataset S5: Contigs of the real human gut dataset.

Supplemental Dataset S6: Scaffolds of the real human gut dataset.

Each file is a `7z` archive and can be extracted, e.g. using command: `7za x archive.7z`
Each extracted directory contains a `readme.txt` file describing all the files contained in the directory.

Software installation

Follow the installation instructions and go through the tutorial. Both can be found here: https://github.com/algbioi/ppsp/wiki

Real datasets

Follow the tutorial:

- Create the pipeline directory in directory: `/apps/pps/tests`
- Use configuration file:
  `/apps/pps/tools/config_ppsp_vm_refNCBI20121122_example.cfg`
  as a template (i.e. copy this file and modify it appropriately).
- Make sure, you set the following parameters in the configuration file:
  `pipelineDir`

inputFastaFile
inputFastaScaffoldsFile
scaffoldsToContigsMapFile

- Run the pipeline using command:

```
ppsp -c CONFIGURATION_FILE -n -g -o s16 mg -t -p c s v -r -s
```

- Analyze the results as described in the tutorial.

Simulated datasets

Follow the tutorial:

- Create the pipeline directory in directory: /apps/pps/tests
- Use configuration file:
  /apps/pps/tools/config_ppsp_vm_refNCBI20121122_example.cfg
  as a template (i.e. copy this file and modify it appropriately).
- Make sure, you set the following parameters in the configuration file:
  pipelineDir
  inputFastaFile
  referencePlacementFileOut
  excludeRefSeqRank (e.g. excludeRefSeqRank=species)
  excludeRefMgRank (e.g. excludeRefSeqRank=strain)
- Run the pipeline using command:

```
ppsp -c CONFIGURATION_FILE -n -g -o s16 mg -t -p c -r -s
```

- Analyze the results as described in the tutorial
